# Saccharopine and α-Aminoadipic Acid as Potential Biomarkers for Mitochondrial Stress in Neurons

**DOI:** 10.3390/cells15191804

**Published:** 2026-10-02

**Authors:** Anna-Katharina Ückert, Anja Land, Regina Pape, Mahshid Alimohammadi, Ivano Amelio, Marcel Leist

**Affiliations:** 1 In Vitro Toxicology and Biomedicine, Department Inaugurated by the Doerenkamp-Zbinden Foundation, University of Konstanz, 78457 Konstanz, Germany; anna-katharina.ueckert@uni.kn (A.-K.Ü.); anja.land@uni.kn (A.L.); regina.pape@uni.kn (R.P.); mahshid.alimohammadi@uni-konstanz.de (M.A.); 2 Systems Toxicology, University of Konstanz, 78457 Konstanz, Germany; ivano.amelio@uni-konstanz.de

**Keywords:** mitochondrial respiration, amino acid analyzer, metabolomics, berberine, 1-methyl-4-phenylpyridinium, tebufenpyrad, MG-132, aerugine

## Abstract

**Highlights:**

**What are the main findings?**

- Mitotoxicants (complex I inhibitors), but not other toxicants, increase neuronal saccharopine levels.
- Absolute concentrations of amino acids, and their changes over time, were determined in neuronal cell cultures.

**What are the implications of the main findings?**

- Saccharopine should be further explored as a potential biomarker for disturbed mitochondrial function in intoxications and neurodegenerative disease.
- The data on cellular amino acid concentrations are an important source for systems biology modelling and general understanding of cellular phenotypes.

**Abstract:**

Redox stress and subsequent metabolic reprogramming are well-known consequences of neuronal exposure to mitochondrial toxicants. Here, we investigated, using human neuronal cultures, how such changes are quantitatively reflected in the patterns of cellular and extracellular amino acids. We asked whether these alterations may yield a mechanistically anchored biomarker for the type of neurotoxicity resulting from the impairment of the respiratory chain. Using a targeted metabolomics approach, we quantified intra- and extracellular amino acid pools and their temporal dynamics. Data were obtained from differentiated LUHMES neurons exposed to the complex I inhibitors berberine, 1-methyl-4-phenylpyridinium (MPP^+^), and tebufenpyrad, as well as to the non-mitochondrial toxicants MG-132 (proteasome inhibitor), RSL3 (glutathione peroxidase 4 inhibitor), and aerugine (fungal oxidative toxicant). The reductive stress, caused by mitochondrial inhibitors, was reflected in a characteristic and specific re-routing of amino acid metabolism, including increased Asn/Asp and Gln/Glu ratios and an upregulation of the electron acceptor Pro in neurons. Most conspicuous were the pronounced and reproducible increases in the lysine catabolites saccharopine (sacc) and α-aminoadipic acid (α-AAA). These two potential biomarkers of neuronal mitotoxicity were not altered by other types of toxicants. Moreover, the extent of concentration changes in sacc and α-AAA roughly correlated with the damaging effects of mitotoxicants, when assay conditions were (exposure period and exposure concentration). Our data provide an exemplary reference map of absolute amino acid concentrations in human cells and their culture medium. Moreover, sacch and α-AAA emerge as candidate biomarkers for neuroactive mitotoxicants and potentially for pathologies associated with impaired neuronal mitochondrial function.

## 1. Introduction

The inhibition of a single cellular function (e.g., mitochondrial respiration) may have multiple direct and indirect effects on cellular metabolism. These metabolome changes may modulate, facilitate or cause the final toxic outcome. Moreover, the pattern of changes can inform on the toxicant’s mode of action. Finally, certain features of metabolome changes could also serve as biomarkers of toxicity [1,2,3,4,5,6,7].

The comprehensive analysis of metabolites in biological systems is often termed metabolomics [3]. It has been utilized in drug discovery to investigate toxicity mechanisms. It also offers solutions to overcome the limitations of existing methods for determining toxicity pathways [8]. The potential of metabolomics approaches to identify novel, unexpected changes, often linked to amino acid metabolism, has been demonstrated in several studies. For instance: (i) An integrated metabolomics and network toxicology approach was used to investigate the neurotoxic mechanisms of bisphenol A (BPA). Glu metabolism was identified as a hitherto unknown contributor to BPA-induced neurotoxicity in zebrafish embryos [9]. (ii) The hepatotoxicity of okadaic acid has been linked to a disruption of Cys metabolism and lipolysis regulation [10]. (iii) Untargeted metabolomics analyses of gelsenicine poisoning revealed disruptions in energy metabolism, with core metabolites like malate, Glu, and aspartate emerging as potential biomarkers of toxicity [11].

The gap between mechanistic studies and potential regulatory applications has been addressed by several large-scale metabolomics initiatives in toxicology. For instance, data on the plasma metabolome of rats have been obtained for hundreds of compounds. They have been used for the construction of the MetaMap(^®^)-Tox database, and metabolite patterns predictive of several target organs have been identified [12]. Grouping of compounds based on metabolomics similarities is considered very promising for risk assessment [13]. In the MATCHING ring trial, 6 laboratories analyzed the metabolic changes elicited by 8 candidate toxicants and were able to reproducibly group them according to their metabolic patterns [6]. Using such information, together with other databases, the MTox700+ metabolite panel was assembled as a publicly available biomarker panel (722 metabolites) for adverse outcomes and associated molecular pathways in toxicology [14]. For the application in regulatory toxicology, standardization requirements have been discussed [15], and studies on the repeatability of novel findings are urgently required.

Changes in the metabolome do not only occur when toxicants affect metabolic enzymes or relevant transporters directly. It has been demonstrated in rodent studies that toxicants that are considered to primarily alter electrophysiological properties, like the pesticides chlorpyrifos or deltamethrin, have significant metabolic effects on lipid and amino acid metabolism [16,17]. Studies on human cells (SH-SY5Y) have shown that other pesticides (strobilurin and triazole fungicides) have severe consequences on oxidative stress, lipid and amino acid metabolism [18,19].

One of the most prominent classes of “metabolic toxicants” are inhibitors of the mitochondrial respiratory chain [20]. Such compounds can selectively induce neuronal cell death [21,22]. Some of them are explicitly used to develop models for human neurodegenerative diseases: (i) 1-methyl-4-phenyl-1,2,3,6-tetrahydropyridine (MPTP) generates a pathology related to Parkinson’s disease [23], (ii) 3-nitropropionic acid and methylmalonic acid trigger striatal degeneration, as, e.g., found in Huntington’s disease [24,25]. Based on such studies, the potential role of mitochondrial inhibitors as plant protection products is considered a risk factor for Parkinson’s disease [26,27,28].

An important tool for modern risk assessment are adverse outcome pathways (AOPs) [29,30,31] that deliver mechanistic information on the causal links between molecular initiating events (MIE; e.g., binding of a toxicant to its target) and an adverse outcome (AO; final pathological manifestation) of regulatory concern. They provide the biological context to facilitate the development of integrated approaches to testing and assessment (IATA) for various regulatory purposes [32,33,34]. An AOP-informed next-generation risk assessment (NGRA) [35,36] aims to quantify all important steps, also called key events (KEs). Particularly relevant for respiratory chain toxicants is AOP:3 [27], which describes the linkage between inhibition of complex I (cI) of the mitochondrial respiratory chain and motor deficits as in Parkinsonian disorders. Experiments conducted with the cI inhibitors rotenone and MPTP reveal a significant concordance in the dose–response relationships between MIE, KE and AO [27]. This has been formalized in mathematical models describing AOP:3 [37]. Metabolic profiling indicated that, especially, amino acid metabolism is deregulated after exposure to mitotoxicants/cI inhibitors [38].

While AOPs focus on linear sequences of molecular/cellular events, they tend to neglect the indirect, secondary network effects on metabolism. For instance, cI inhibitors may lead to an increased cellular NADH/NAD^+^ ratio, associated with reductive stress [39,40,41,42,43]. This has widespread neuronal consequences, e.g., on various amino acid metabolite pathways [38] and on protein aggregation [44]. In human neurons, it has been shown that a block of a degradation pathway, as for Lys or branched-chain amino acids, may lead to the accumulation of products that have toxic or modulatory effects in other pathways. Accordingly, a block of cI not only reduces downstream ATP production, but it also increases the levels of metabolites like saccharopine (sacc). Such an accumulation upstream of the bottleneck favours new pathways and alters metabolite pools sometimes “far away” from the inhibited reaction. A typical example is upregulation of transsulfuration as a consequence of increased oxidative stress [38,45].

In line with this, it has been observed that toxicants with similar inhibitory effects on cI may show largely different toxicity responses [21,38,46]. To better understand this, a focus on metabolome changes may be helpful. As amino acid metabolism connects energy metabolism and biosynthetic processes (see also Supplementary Discussion), we provide a solid database on amino acid contents in the chosen model system at the beginning of our study. As a model system, we chose LUHMES neurons, which have been used previously for a broad range of neurotoxicity studies [47,48,49,50], especially in the area of Parkinsonian-related neurodegeneration [38,45,51,52,53,54,55]. Key data on AOP:3 have been generated in this system, and first indications of widespread metabolic changes upon exposure to mitotoxicants were obtained [27,38]. Now, we provide a broad overview of cellular amino acid changes, and we connect them to altered levels in the extracellular medium. On this basis, alterations by various mitochondrial and non-mitochondrial toxicants are described. The two Lys metabolites, sacc and α-aminoadipic acid (α-AAA), were further explored, as an earlier study suggested unexpected changes in these less-known metabolites [38]. To explore whether sacc and/or α-AAA may serve as potential biomarkers of neuronal mitotoxicity, we compared their changes after exposure to mitochondrial toxicants and non-mitochondrial toxicants. The outcome suggests that the metabolites deserve further profiling as potential biomarkers.

## 2. Materials and Methods

### 2.1. Materials and Chemicals

Dibutyryl cyclic adenosine monophosphate (db-cAMP), fibronectin, poly-L-ornithine hydrobromide (PLO), tetracycline, 5-sulfosalicylic acid, berberine chloride, tebufenpyrad, 1-methyl-4-phenylpyridinium (MPP^+^) iodide, narciclasine, antimycin A, oligomycin, RSL3, dimethyl sulfoxide (DMSO), methanol, amino acids and L-2-aminoadipic acid were purchased from Sigma (Steinheim, Germany). Recombinant human fibroblast growth factor 2 (FGF-2) and recombinant human glial cell-derived neurotrophic factor (GDNF) were purchased from R&D Systems (Minneapolis, MN, USA). All cell culture reagents were purchased from Gibco/Fisher Scientific (Hampton, NH, USA) unless otherwise specified. Buffers and columns for amino acid analysis were purchased from Sykam (Fürstenfeldbruck, Germany). Saccharopine was purchased from Toronto Research Chemicals (Vaughan, ON, Canada). Hoechst H-33342 was purchased from Thermo Fisher Scientific (Darmstadt, Germany). Calcein-AM was acquired from AAT Bioquest (Pleasanton, CA, USA). Ethanol was purchased from Roth (Karlsruhe, Germany). 2-Propanol was purchased from VWR Chemicals (Darmstadt, Germany). MG-132 was purchased from SelleckChem (Köln, Germany). Aerugine was synthesized by the group of Thomas Böttcher [56].

### 2.2. LUHMES Cell Culture

LUHMES cells are a sub-clone of a neural precursor cell line, which was derived > 25 years ago from ventral mesencephalic tissue of an 8-week-old female human embryo. The cells, with ATCC number CRL-2927, have a normal karyotype and primary gene sequence. They are modified by a conditionally active v-myc transgene with a known insertion site and controlled by a tet-off system. They have been short tandem repeat (STR) authenticated, and they have been considered to pose no ethical concerns as an established cell line [57]. Cell culture followed established procedures [58,59,60]. In brief, cells were grown in standard cell culture flasks pre-coated with 50 µg/ml PLO and 1 µg/ml fibronectin in water for at least 3 h at 37 °C. The maintenance culture was kept in proliferation medium consisting of advanced DMEM/F12 with 2 mM L-Gln, 1x N2-supplement, and 40 ng/ml FGF-2. The cells were incubated at 37 °C with 5% CO_2_ and passaged thrice a week when reaching 75–90% confluence. They were used up to passage 18. For differentiation, the medium was changed to differentiation medium consisting of advanced DMEM/F12 supplemented with 2 mM L-Gln, 1 mM db-cAMP, 1 µg/ml tetracycline and 2 ng/ml GDNF on day of differentiation 0 (d0).

### 2.3. Neurite Outgrowth Assay

The assays were performed as described previously [38,49,54,60,61,62]. In brief, 30,000 cells per well were seeded on d2 in differentiation medium into standard pre-coated 96-well plates. They were incubated for 1 h at 37 °C and 5% CO_2_ before treatment. 24 h after treatment, the Calcein-AM/Hoechst readout was performed.

### 2.4. Neurite Area and Viability Readouts

Neurite area (NA) and viability (V) were assessed as described previously [61]. In brief, cells were stained with 1 µM calcein-AM and 1 µg/ml Hoechst H-33342 for 30 min at 37 °C and 5% CO_2_. Image acquisition was performed using an Array-Scan VTI HCS Reader (Cellomics, Pittsburgh, PA, USA) equipped with a Hamamatsu ORCA-ER camera. 10 fields per well were imaged with 2 channels at 20× magnification (2 × 2 pixel binning). Excitation/emission wavelengths of 365 ± 50/535 ± 45 were used for Hoechst H-33342 detection in channel 1, and 474 ± 40/535 ± 45 were used for calcein-AM detection in channel 2.

Nuclei were identified in channel 1 depending on intensity, area, size and shape. Their outlines were expanded by 3.2 µm to define a virtual cell soma area (VCSA), which was bigger than the average cell soma to prevent false-positive NAs. All calcein-positive pixels were defined as viable cellular structures (VCSs). The NA was automatically calculated by excluding the VCSAs from the VCSs. Furthermore, all nuclei co-localizing with VCSs were defined as alive.

### 2.5. Determination of Cell Volume

The volume of LUHMES cells was determined with two methods [54]: (1) A large number (50 million) of cells were centrifuged mildly (300× *g*, 5 min) into a 1.5 mL reaction tube, and the supernatant medium was removed. The volume of the pellet was determined by careful comparison to tubes filled with defined amounts of water, using weight and meniscus height as readouts. This method resulted in a calculated single-cell volume of V = 1370 µm^3^ (=1.4 pL). (2) Proliferating LUHMES cells were passaged as usual and seeded into coated 24-well plates. After a brief attachment period, brightfield images were taken, and the diameters (d) of 22 cells were measured. It was assumed that cells under these conditions took the shape of a hemisphere. Based on the measured diameters, the predicted volume was V = 1625 µm^3^ (=1.6 pL). The results were averaged to a volume of V = 1500 ± 180 µm^3^, i.e., about 150 nL for 100,000 LUHMES cells.

### 2.6. Amino Acid Analysis

Amino acid analysis was performed exactly as described by Delp et al. [49]. LUHMES cells were seeded on d2 into coated 10 cm dishes at a density of 9 × 10^6^ cells per dish in 10 mL of differentiation medium without db-cAMP and GDNF. They were treated as indicated and then harvested on d3. For the quantification of extracellular amino acid concentrations, medium samples were prepared: medium (780 μL) was mixed with 10% 5-sulfosalicylic acid (195 μL); tubes were shaken for 30 min at 4 °C and 1400 rpm in an Eppendorf Thermomix (Hamburg, Germany). After centrifugation for 30 min at 4 °C and 20,000× *g*, the supernatant was diluted 1:2 with 120 mM manufacturer-supplied sample dilution buffer (pH 2.2) (Sykam, Fürstenfeldbruck, Germany).

For the quantification of intracellular amino acid concentrations, cell lysates were prepared: The cell culture medium was aspirated, and the cells were washed once with phosphate-buffered saline (PBS). Then, 900 µL of ice-cold 50% (*v*/*v*) methanol was added; the cells were scraped off and transferred into a reaction tube. Another 400 µL of ice-cold 50% methanol was added to the cell dishes for washing. They were transferred into the same reaction tube. The sample tubes were shaken for 30 min at 4 °C and 1440 rpm in an Eppendorf Thermomix (Hamburg, Germany). Then, they were centrifuged for 30 min at 4 °C at 20,000× *g*. The supernatant was transferred into a fresh reaction tube. The pellet was resuspended in 400 µL of ice-cold 50% methanol, shaken for 20 min at 4 °C and 1440 rpm, and centrifuged for 20 min at 4 °C at 20,000× *g*. The supernatant was added to the previously collected supernatant, lyophilized in a SpeedVac concentrator and reconstituted in 135 µL of 5-sulfosalicylic acid (2%) containing 10 µM norleucin as an internal control. After shaking for 30 min at 4 °C and 1400 rpm, samples were centrifuged for 30 min at 4 °C and 20,000× *g*. The resulting supernatant was removed, and 125 μL was transferred into a snap-ring sample vial for amino acid quantification. Analysis procedure: The individual amino acids were separated and quantified using a Sykam S433 amino acid analyzer (Sykam, Fürstenfeldbruck, Germany) with a lithium-based anion exchange column (7 µm diameter, 10% cross-links, cat# 5125022) and post-column derivatization with ninhydrin (Figure S1). Elution was performed with buffers of varying ion strength and increasing pH, and a temperature gradient (Figure S2). The ninhydrin-amino acid reaction products were quantified at 570 nm and 440 nm, depending on the amino acids (Figure S1). The peak areas were determined using the ChromStar version 7.0 software (SCPA, Weyhe-Leehste, Germany) and converted into concentrations using defined standards. The original method was adapted to the quantification of sacc and α-AAA as part of this study (therefore described in the results chapter). Briefly, a new separation method was developed and applied for the quantification of α-AAA. This was necessary to separate it better from Cys (reduced cysteine). Sacc was identified together with all other amino acids. It partially overlapped with the cystine (oxidized form of cysteine) peak. This was no problem, as cystine was virtually absent from cell samples, and detection on two wavelengths allowed for a fully quantitative analysis. The method was not formally validated by a second, orthogonal method (e.g., mass spectrometry). However, the results obtained here are highly correlated with an earlier study that used MS for the quantification of Sacc and α-AAA (described in [39]).

### 2.7. Data Analysis and Further Information

Unless stated otherwise, all data are expressed as means ± SEM of at least three biological replicates. The methods for statistical analysis are indicated in the respective figure legends. To allow readers to use other forms of display and statistical analysis, all data are provided in a machine-readable format. The original data used for the generation of figures, heatmaps and other displays are available in the detailed Supplementary Information (SupplementaryData.xlsx). The file is also available in Zenodo [63]. Some further background information—data and literature [50,55,64,65,66,67,68,69,70,71,72,73,74,75,76,77,78,79,80,81,82,83,84,85,86,87,88,89,90] as well as additional displays have been compiled in the Supplementary Information PDF file.

## 3. Results and Discussion

### 3.1. Amino Acid Content in Cultures of Human Neurons

To obtain data on the “normal” amino acid content of cultured neurons, we used an exemplary human neuronal model, based on LUHMES cells differentiated for three days (d3) [50,57,91]. Cell extracts were generated from non-stimulated neuronal cultures, and 45 amino acid-related metabolites (including urea and GSH) were quantified. After separation by HPLC and continuous post-column derivatization of amino groups with ninhydrin, absorbance was used as the readout (Figure S1). The quantification was based on a calibration of peak areas by reference standards. This method (named here AA-quantification (AAQ)) yielded data with high reproducibility: there was <5% variation across analytical runs [92,93,94], even when samples from different experiments were compared.

**Figure 1:**
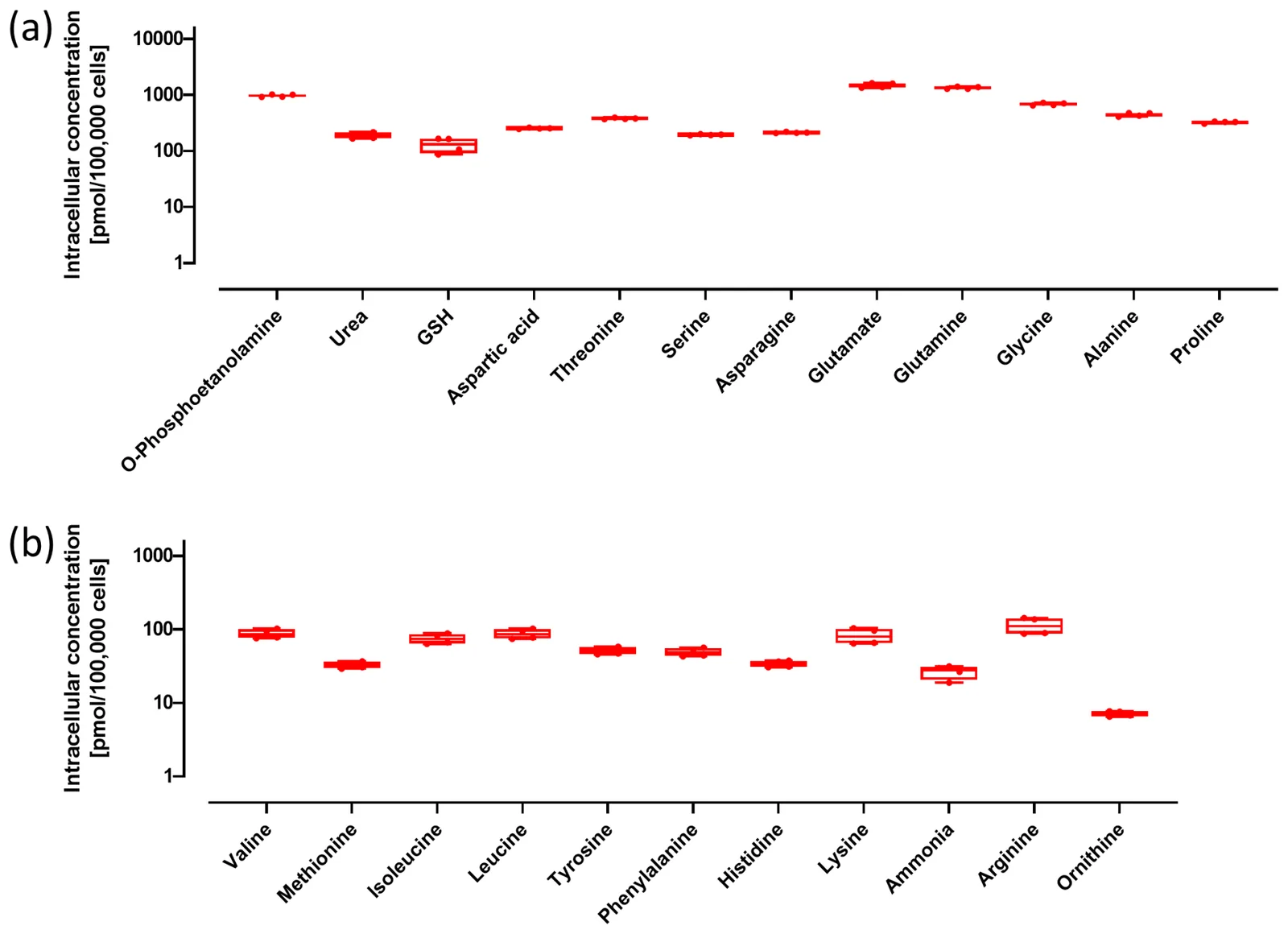
Intracellular amino acid concentrations of LUHMES cells. (**a**,**b**) LUHMES cells were plated on d2 into 10 cm dishes and incubated for 24 h at 37 °C and 5% CO_2_ before cells were lysed and their amino acid composition was analyzed. Data are presented as box-and-whisker plots with a total of four independently prepared replicates. More amino acids can be seen in Figure S3.

To calculate the intracellular metabolite concentration from the cell lysate data (Figure 1 and Figure S3), these were normalized to the known cell volume of 1500 ± 180 nL for 10^6^ LUHMES cells [54]. The relative abundances of amino acids differed by more than two orders of magnitude as shown by the following examples: Glu, the most abundant amino acid in our model, was found at an intracellular concentration of 9.9 ± 0.9 mM (15 ± 1.4 nmol per 10^6^ cells); concentrations of Leu (a typical mid-range amino acid) were 580 ± 90 µM (870 ± 14 pmol per 10^6^ cells) and ornithine (48 ± 4 µM; 70 ± 6 pmol per 10^6^ cells) was the least abundant amino acid (Figure 1 and Figure S3). Notably, we use the term ‘amino acid’ here in a chemical sense, referring to all compounds that can be detected and quantified by AAQ on the basis of a ninhydrin reaction. This includes most proteinogenic amino acids, but also ornithine, GABA and saccharopin as biochemically relevant non-proteinogenic amino acids.

Reference values (e.g., from a database or from a panel of publications) on intracellular amino acid levels in cultured neurons are not readily available. The use of brain tissue data also faces several challenges [95]. For instance, large differences in the amino acid patterns (relative ranking) and absolute amounts have been observed between various brain regions and also between corresponding regions of rat and human brain [96]. This may be due to differences in the extracellular environment and in the contribution of glial cells.

Data on cultured cells also have considerable variability: A comparative study of cultured cells (A549 vs. U87) found that the relative abundances differed [97]. On average, this study measured 5–10 times higher levels than a detailed study on HeLa cells in different media [98]. Most likely, some of the difference in absolute concentrations is related to technical difficulties in determining the cell volumes. The cell volume data used in our study are based on two different methods. They were very consistent, even when repeated by different operators. The absolute concentrations determined on this basis are mostly in the range of concentrations found for HeLa cells earlier. Some comparisons show that in all studies (HeLa, LUHMES, A549, U87), the most abundant amino acids were Ala, Gln, Glu and Gly, followed by Pro. The absolute levels were in the 8–12 mM range in the study on A549/U87, and mostly in the 0.3–2 mM range for HeLa and LUHMES cells. Only Glu & Gln were in the 8–10 mM range in LUHMES. In all studies, Lys, Met and His showed very low abundances. Differences between cells/studies became most obvious in the intermediate range. In A549 cells, the branched-chain amino acids Ile, Leu and Val were more abundant than Asp and Thr. This ranking was reversed in U87. In our study, the branched-chain amino acids (about 500 µM) were less abundant than Asp and Thr (about 2 mM). The ranking in LUHMES cells agreed with findings in HeLa cells.

### 3.2. Amino Acid Changes in the Culture Medium of Neurons

**Figure 2:**
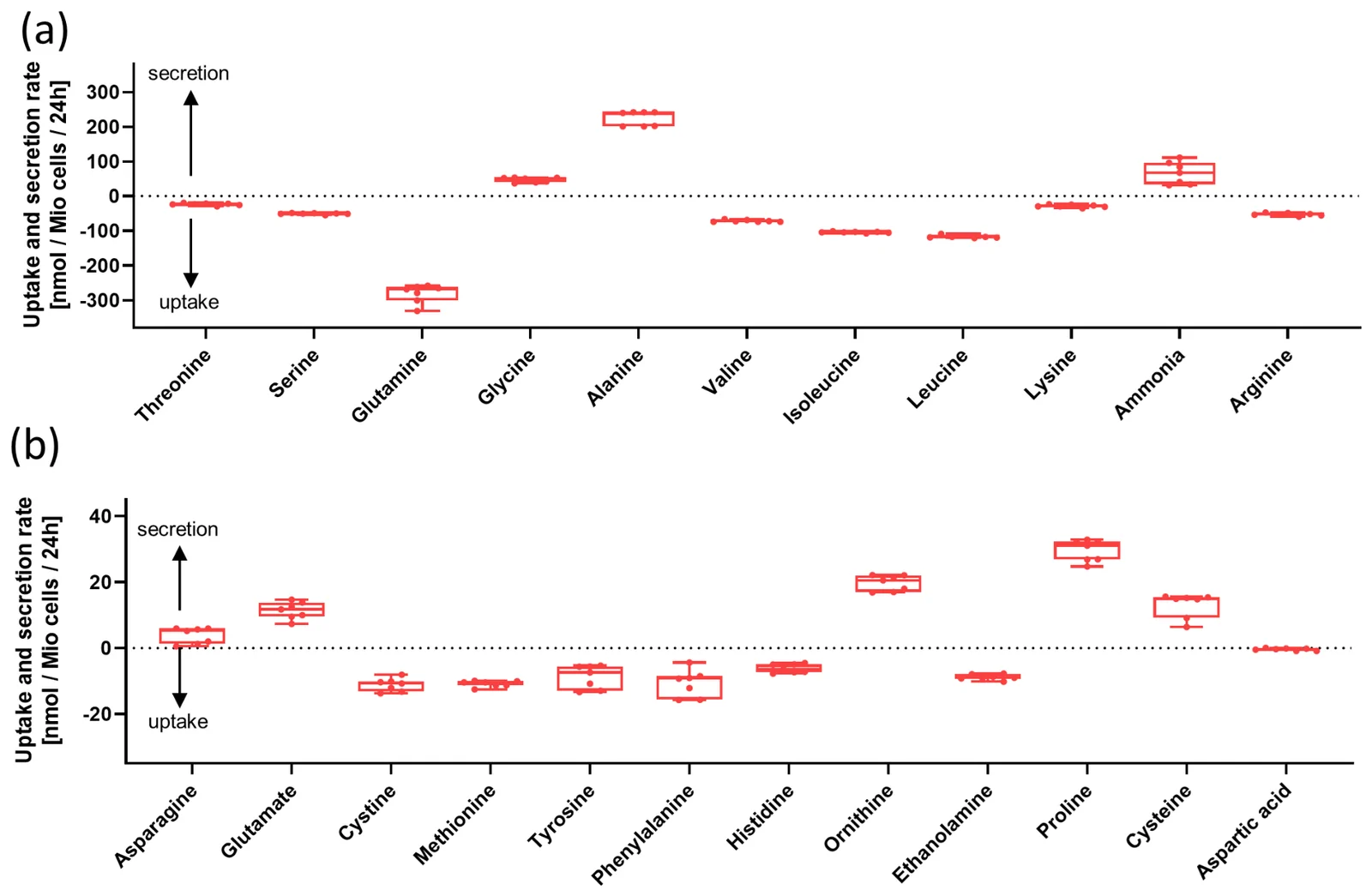
Amino acid uptake and secretion rates of LUHMES cells. (**a**,**b**) LUHMES cells were plated on d2 into 10 cm dishes and incubated for 24 h at 37 °C and 5% CO_2_, before the amino acid composition of the medium was analyzed. Uptake and secretion rates were calculated from the difference in the amino acid concentrations measured in medium with and without cells. Data are presented as box-and-whisker plots with a total of seven independently prepared replicates.

Having determined the intracellular concentrations of amino acids in cultured neurons, we were interested in how the cells affected the metabolite pattern of their surroundings, i.e., of the cell culture medium. We therefore analyzed media incubated with and without cells. We assumed that differences measured under these two conditions were mainly due to cellular uptake and secretion. On this basis, we here interpreted and described losses and gains in the medium compartment as “cellular uptake and secretion rates” (Figure 2). According to this, Ala was the amino acid that increased most strongly in the medium. We calculated a secretion rate of 224 ± 21 nmol per 10^6^ cells per 24 h. This is also in the range determined in a metabolic flux model of non-differentiated LUHMES cells [49]. The biochemical rationale is that cells cultured in the presence of high glucose concentrations predominantly use glycolysis for energy production and thus produce large amounts of pyruvate. They also require a nitrogen sink (disposal of NH_3_), as NH_3_ is generated during the use of amino acids for biosynthesis of lipids (neurite growth) and as input of the citric acid cycle. Ala is produced (indirectly) from ammonia and pyruvate and conveniently secreted as a “waste product” of metabolism.

Glu (11 ± 3 nmol per 10^6^ cells per 24 h) and ornithine (20 ± 2 nmol per 10^6^ cells in 24 h) secretion rates were lower, but both amino acids still increased in the medium. They likely served as a nitrogen sink. For instance, Glu is generated from many amino acids via transaminase steps and conversions in the citric acid cycle.

Gln was the amino acid most clearly depleted from the medium, with an assumed cellular uptake rate of 280 ± 27 nmol per 10^6^ cells per 24 h. Leu (116 ± 4 nmol per 10^6^ cells per 24 h) also exemplifies an amino acid that was strongly depleted from the medium and thus is assumed to be taken up by cells. Histidine exemplifies an amino acid that changed very little (loss of 6 ± 1 nmol per 10^6^ cells per 24 h) (Figure 2). These data give an indication of amino acid fluxes to be expected in LUHMES or possibly other neurons. This exemplary data set may not be transferred to other cultures, but it showcases the dynamic state of cell cultures and their media. Alterations of medium constituents (expected to occur in most cultures) are associated with changes in metabolic and biosynthetic supply of cells, and are potentially important for data interpretation and systems biology modelling.

Our experiments took into account that stability of amino acids may play a role in altered abundances. We measured the changes also in medium without cells, and they were negligible, with the exception of Cys, which tends to oxidize. The assumption of simple fluxes in and out of cells may also be a simplification, and more detailed data may be obtained by flux analysis using isotope-labelled amino acids. However, our data on significant changes in the overall medium contents are valid and robust. They suggest that for the interpretation of cell-based metabolomics analyses, it may be necessary to obtain information on both intracellular and extracellular metabolite pools.

To provide an easily accessible overview of the relative losses of amino acids from the medium (assumed to equal cellular uptake), the differences were calculated as a percentage of the original medium content. This yielded the percentage of internalization for each amino acid (Table 1). For instance, only a low percentage of histidine was internalized (4.2% ± 0.8%). This concurs with its low uptake rate. However, “% loss from medium” and “size of uptake rate into cells” were not always correlated. For instance, Leu had a much lower uptake rate (60%) than Gln. But a higher percentage of Leu (23.4% ± 0.8%) was internalized than for Gln (13.6% ± 1.2%). This is explained by the lower medium concentration of Leu (compared to Gln). Information on the relative uptake of amino acids from the medium can be used in several contexts, e.g., to optimize medium composition or medium exchange schedules. For toxicological studies, the change in basal uptake or secretion rates by toxicants may provide information not easily obtained from other assays.

Our data show that there is a reciprocal relationship between amino acid contents in cells and the medium. While it is clear that cells use the amino acid pool of the surrounding medium, they also change this environment. One may ask what this environment should most typically be. However, typical environments in the body also differ from one another and over time (e.g., portal vein vs. other vascular beds in the postprandial vs. fasting phases, or under low and high physical workloads). In cell cultures, the cellular content of branched-chain amino acids has been shown to strongly depend on the medium. The situation is even more extreme for Gln: the medium supply may affect cellular contents up to 50-fold [98]. Our cell culture medium contained initially 2 mM Gln (like most media), but this was reduced over time by cellular uptake. Typical plasma or CSF levels are in the range of 0.5 mM [99].

Concerning neurons, one needs to consider that their environment is not plasma, but rather CSF. Extensive studies have shown that the amino acid concentrations in CSF are often lower than in plasma. This applies in different ways to individual amino acids. For instance, Arg, Asp and Thr have relatively high CSF levels (20–50% of plasma levels), while Ala, Val and Leu have a CSF/plasma ratio of <0.1, and Cys, Gly, Glu and Trp have particularly low concentrations in CSF. With such comparisons, one needs to bear in mind that some amino acids are supplied to neurons by surrounding astrocytes (e.g., Glu, Cys) through complex transcellular biochemical pathways [100,101]. The studies described here give some orientation and background on our findings. The dearth of available information in this area also suggests that more data on cellular metabolites and their exchange with the cell environment are desirable. This applies both to in vivo situations and to the ever-increasing number of in vitro systems (including organoids and microphysiological systems) used in biomedical research.

### 3.3. Modification of AAQ to Quantify Sacc and α-AAA

The method used here for AAQ not only allows the assessment of “standard” amino acids (the conventional set of proteinogenic L-amino acids), but also of a broader range of primary and secondary amines. We were particularly interested in the Lys degradation products saccharopine (sacc) and α-aminoadipic acid (α-AAA), as these two compounds have been suggested earlier as potential biomarkers of neurotoxicity triggered by inhibitors of the mitochondrial respiratory chain [38]. We were interested here in confirming original findings by an alternative, highly quantitative method. Moreover, we were interested in more information on the concentration- and time-dependence of such putative biomarkers of mitochondria-related neurotoxicity, as well as on the specificity against other toxicants.

**Figure 3:**
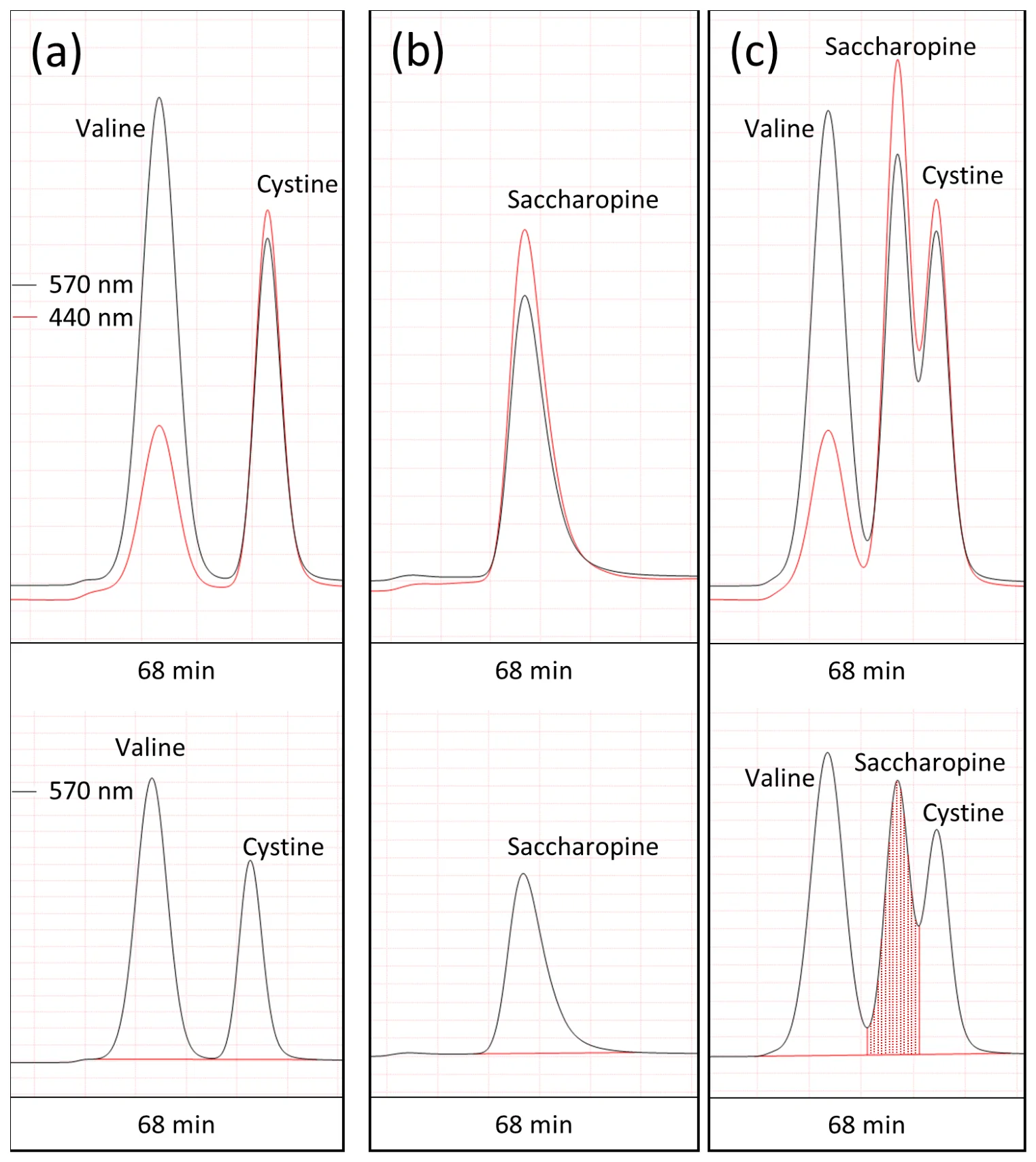
Detection and quantification of the metabolite saccharopine. Relevant chromatogram sections (eluting at about 68 min) are shown for a standard (calibration) mixture of amino acids. A mix of amino acid standards was analyzed without saccharopine (**a**), for saccharopine alone (100 µM) (**b**), and for a mix of amino acid standards including 100 µM saccharopine (**c**). Each amino acid of the mixture had a concentration of 100 µM (only cystine was used at 50 µM). Peaks are labelled with the respective amino acids. The chromatogram detected at an absorption of 570 nm is shown in black. The chromatogram recorded at 440 nm is shown in red. (**a**) A volume of 100 µL was injected. An example of the full chromatogram is shown in Figure S4. The corresponding method (AAQ) is detailed in Figure S2. The upper row shows chromatogram traces used for peak identification. The lower row shows the information used by the software for peak quantification. (**a**,**b**) show that saccharopine runs in the area where Val and cystine run. (**c**) Saccharopine can be distinguished from cystine and Val in a mixed standard. The quantified peak is highlighted with red shading.

Some adaptations were required, as the method for amino acid quantification used earlier in our laboratory did not yield data on the sacc and α-AAA content of samples (Figure S2). The quantification of sacc was solved relatively easily by including this amino acid in the calibration mixture. When spiking sacc onto our reference standard (containing 45 amino acids and amines), it ran close to Val and cystine. However, we found a sufficient separation of the three amino acids (Val, cystine, sacc) to allow a reliable quantification of sacc at 570 nm (Figure 3 and Figure S4). Thus, no modifications of the analysis method (AAQ) as such were necessary. The limit of detection (LOD) allowed by this approach was about 1.5 pmoles/million cells. The limit of quantification (LOQ) was 15 pmoles/10^6^ cells, which translated to a cellular concentration of about 10 µM. The linear range extended to about 800 µM in cells (1.2 nmoles/10^6^ cells).

**Figure 4:**
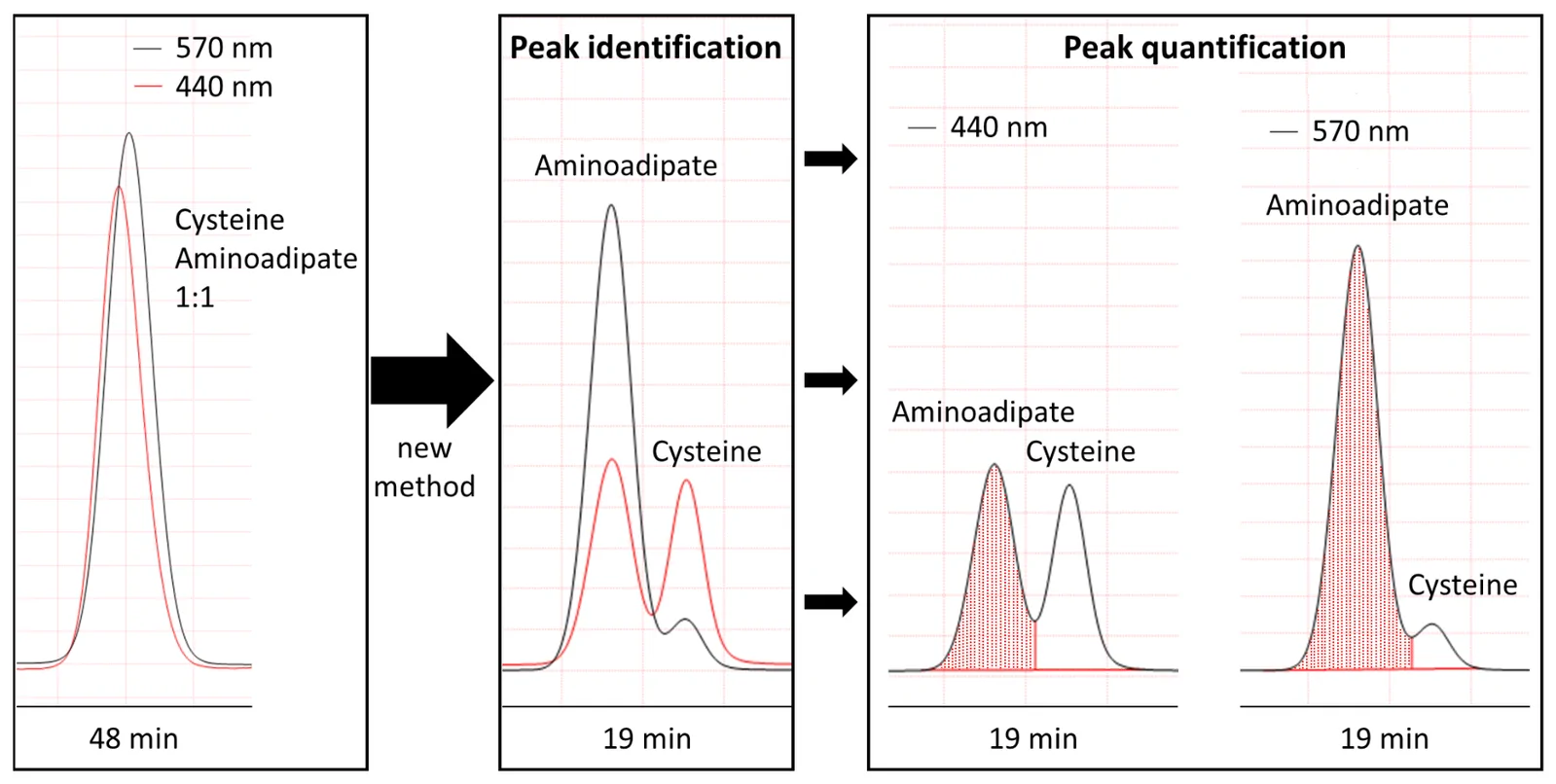
Separation of cysteine and α-aminoadipic acid. In the original “AAQ” method, α-aminoadipic acid and Cys could not be distinguished, as shown in the chromatogram on the left (the full chromatogram is shown in Figure S5). The new “AAQ2” method (detailed in Figure S6) resulted in a separation of the two metabolites after a runtime of 19 min. The absorbance at 570 nm was used to quantify α-aminoadipic acid, whilst Cys was quantified at 440 nm. The α-aminoadipic acid peak is highlighted with red shading at both wavelengths.

For α-AAA, the situation was different. It was already included in the original reference standard. However, its peak overlapped strongly with that of Cys. This made the quantification of these 2 metabolites unreliable (Figure 4 and Figure S5). Taking into account the isoelectric points of the metabolites and their dependence on temperature, we established a second method (AAQ2), which we used exclusively to quantify Cys and α-AAA (Figure S6). Although the peaks partially overlapped when Cys and α-AAA were run together, a reliable quantification was possible by simultaneous measurements at two wavelengths. Cys was quantified at 440 nm, while we used 570 nm for α-AAA. In the experiments performed here, the separation of α-AAA from Cys was practically easy, as Cys was present only at very small concentrations in cell samples after the preparation procedure. This allowed α-AAA to be detected at levels above 0.45 pmoles/10^6^ cells, and to be quantified (LOQ) at levels above 3 µM/4.5 pmoles/10^6^ cells. The linear range extended up to cellular concentrations of about 600 µM. This method (AAQ2) was run in addition to the standard method (AAQ); i.e., two samples were generated and analyzed in two parallel runs (Figure S7).

### 3.4. Toxicant-Induced Changes in Lysine-Related Metabolites

In a previous study, we performed a multi-omics comparison of berberine and several other neurotoxicants. This unambiguously defined berberine as a mitochondrial respiratory chain complex I (cI) inhibitor. Reductive stress and its downstream consequences [39,44] were evident from massively increased NADH changes, and indications of the tricarboxylic acid cycle running in reductive mode. This study suggested a far-reaching deregulation of amino acids in neurons by mitotoxicants. The study also provided the first indication of sacc and α-AAA as being induced by cI inhibitors [38].

The present study was undertaken to put these earlier findings onto a more solid experimental and statistical basis, in the hope of confirming the unexpected findings on some drastic amino acid changes and on potential biomarkers for such a toxicity. Berberine was selected for the present study as a highly selective mitochondrial inhibitor and typical mitotoxicant [102]. We also included MPP^+^ in our small test panel, as it is the best-characterized and most notorious mitochondrial toxicant leading to a specific dopaminergic neurodegeneration [23,27]. We decided not to use the frequently used mitochondrial inhibitor rotenone in this panel, as it was found earlier to not only affect mitochondria, but also the cytoskeleton [38,103]. Instead, we used tebufenpyrad, a well-characterized and specific cI inhibitor [46,104,105,106].

**Figure 5:**
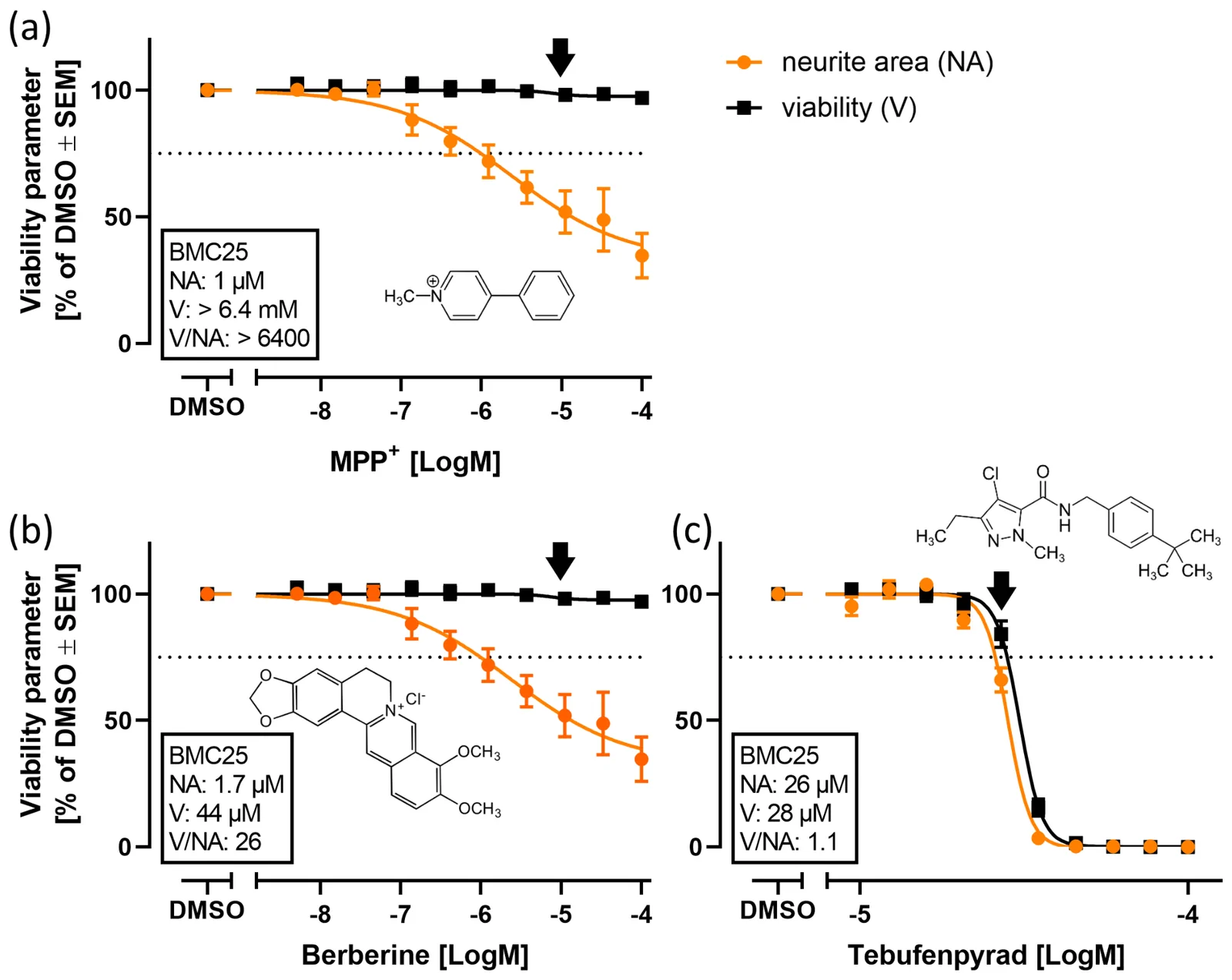
(Neuro)Toxicity of three exemplary complex I inhibitors. Pre-differentiated d2 LUHMES cells were seeded in differentiation medium and incubated at 37 °C, 5% CO_2_ for 1 h. Cells were exposed to serial dilutions of (**a**) berberine, (**b**) MPP^+^ or (**c**) tebufenpyrad (chemical structures are displayed). Cell viability (V; black) and neurite area (NA; orange) were assessed 24 h after treatment by calcein-AM and H-33342 staining and high-content imaging. Data are presented as means ± SEM (see full data set in Supplementary Excel Table). The benchmark concentrations at which the parameters are decreased by 25% (BMC25; dotted line) are given in black boxes. A ratio of BMC25(V/NA) ≥ 4 denotes a specifically neurotoxic compound. The black arrows indicate the concentrations chosen for metabolite analysis.

Initial experiments were designed to identify suitable (non-toxic) concentrations for the metabolomics experiments. Therefore, we determined the concentration-dependent effects of the inhibitors on cell viability and neurite outgrowth (Figure 5). MPP^+^ and berberine reduced neurite outgrowth at significantly lower concentrations than those affecting viability. For further studies, we selected a fixed concentration that did not affect viability but significantly reduced neurite outgrowth (MPP^+^: 10 µM; berberine: 10 µM). Tebufenpyrad reduced viability and neurite outgrowth with a similar potency. Therefore, the highest non-cytotoxic concentration was chosen for further experiments (20 µM).

The viability and neurite area data obtained here (Figure 5) confirm earlier observations [21,22] that inhibitors considered to be relatively specific for cI may show a wide range of cellular effects. Here, cytotoxicity was separated from neurite toxicity: >1000-fold (MPP^+^) or 25-fold (berberine) or not at all (tebufenpyrad). Thus, we were interested in how those differences were reflected by metabolic effects. Importantly, the concentrations selected for metabolite analysis were chosen to avoid overt cytotoxicity. As we found that sacc levels increased at the mitotoxicant concentrations selected as above (below the cytotoxicity threshold), we conclude that sacc increases were more associated with early mitochondrial/neurotoxic stress than with nonspecific metabolite changes caused by cell death.

**Figure 6:**
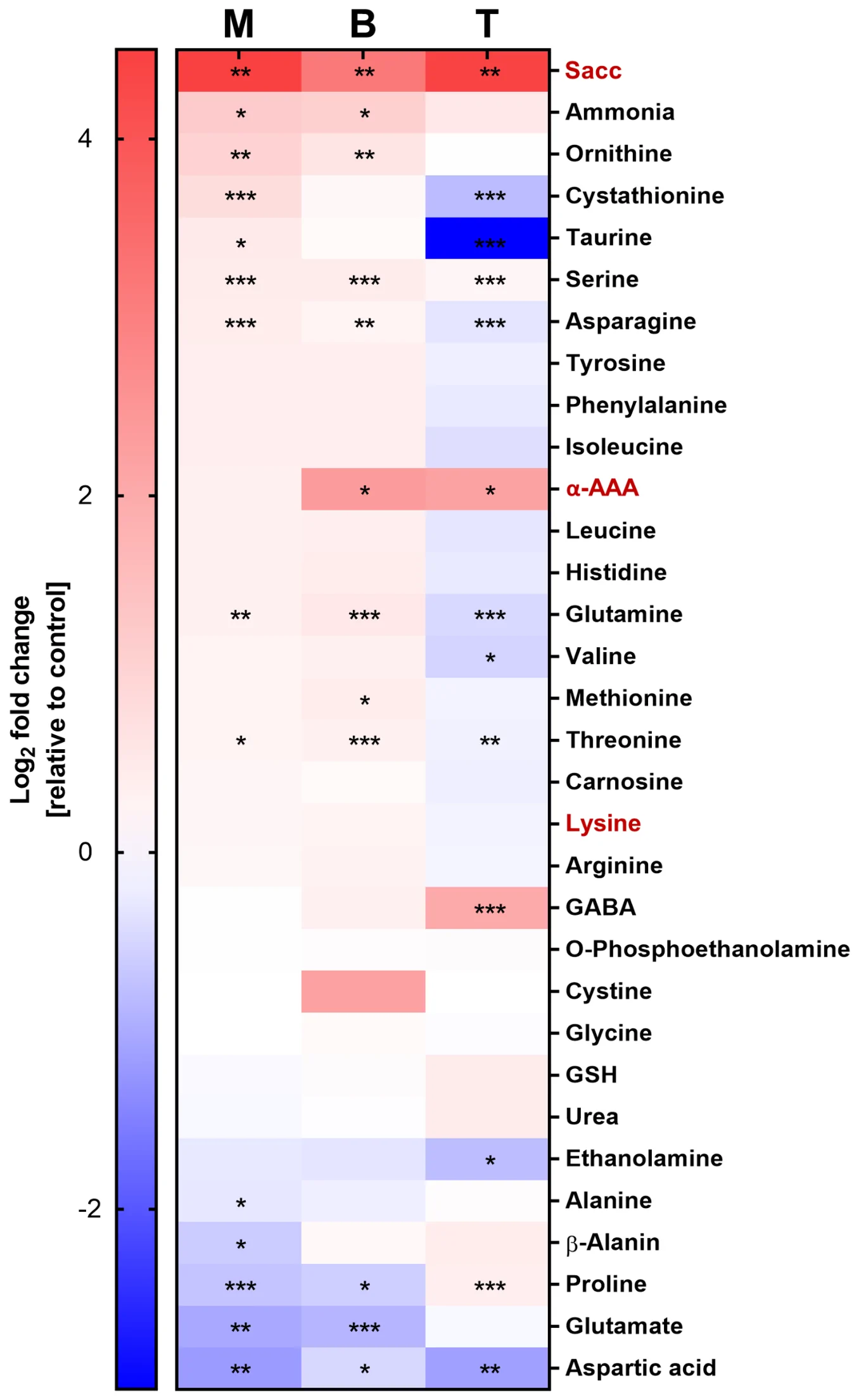
Toxicant-induced changes in intracellular amino acids and related compounds. Pre-differentiated d2 LUHMES cells were seeded in differentiation medium and incubated at 37 °C, 5% CO_2_ for 1 h. They were treated with 10 µM berberine (B), 10 µM MPP^+^ (M) or 20 µM tebufenpyrad (T) for 24 h, before cells were lysed and their amino acid composition was analyzed. AAQ and AAQ2 were used to quantify metabolites in cell lysates. Based on the raw data, the log2-fold changes relative to the untreated control were calculated and presented in the form of a heat map. Negative values (blue) show a decrease in the free amino acid concentration compared to the control; positive values (red) indicate an elevation of the amino acid concentration. All data are compiled in the Supplementary Excel File. For statistical analysis, treated samples were compared to the respective control using a mixed model ANOVA (amino acid x treatment) followed by Dunnett’s multiple comparisons post hoc test. Significance levels are indicated by asterisks (*: adjusted *p* ≤ 0.05; **: adjusted *p* ≤ 0.01; ***: adjusted *p* ≤ 0.001).

To determine toxicant-induced changes at the levels of intracellular amino acids, d2 LUHMES cells were treated with the cI inhibitors for 24 h (Figure 6). Data from these experiments clearly showed that sacc was highly enriched in all treatments with cI inhibitors, and α-AAA was clearly up-regulated by berberine and tebufenpyrad (Figure S8).

As the two metabolites are Lys degradation products generated in the sacc pathway, we consulted mRNA expression data to find out which components of the two major Lys degradation pathways are expressed in LUHMES neurons. All mRNAs, analyzed as in [50], for the necessary enzymes of the sacc pathway were expressed in LUHMES (Figure S9). This is consistent with earlier findings that the major pathway for Lys degradation in the brain proceeds via sacc as the main metabolite [75].

One reason for the accumulation of sacc upon cI inhibition could be a lack of NAD^+^ as a cofactor for the many (five altogether) reductive reactions in the pathway leading from sacc to acetyl-CoA (Figure S10). Such reactions are energetically unfavourable, or nearly impossible under conditions of NADH overload. cI inhibition prevents the oxidation of NADH to NAD^+^, and thus depletes NAD^+^. Thus, sacc accumulation is a typical result of metabolic reprogramming under conditions of reductive stress [39]. The accumulation of α-AAA may have similar reasons (three reductive steps in its degradation pathway). However, the situation is more complex, as NAD^+^ is also required upstream of α-AAA formation, and the reaction balance depends on, e.g., α-aminoadipic acid 6-semialdehyde availability (from other pathways).

### 3.5. Commonalities and Differences in Changed Amino Acid Levels Triggered by Mitochondrial Toxicants

As sacc and α-AAA are part of the network of overall amino acid metabolism, we set out to obtain broader information on related changes before returning to more detailed questions on sacc/α-AAA (see also Supplementary Discussion).

While the metabolic patterns of MPP^+^ and berberine were similar, tebufenpyrad’s metabolic profile clearly differed from that consensus signature. This might be due to the use of this compound at a concentration that was higher than the one required to inhibit cI [105]. As indicated earlier (Figure 5), tebufenpyrad was used at its cytotoxicity threshold, while the other toxicants were used at concentrations that did not affect cell viability (but reduced neurite area). Based on this test concentration choice, the tebufenpyrad concentration was >100-fold higher than the concentration required for cI inhibition, while the other compounds were used in the concentration range coinciding with cI inhibition [46,105,107]. It is therefore likely that tebufenpyrad acted on secondary targets in addition to cI. Such effects are likely to contribute to an altered pattern of changed amino acids.

Based on the above considerations, we regarded the changes triggered by berberine and MPP^+^ as a candidate complex I inhibition-associated signature. Future studies with more inhibitors, and with sac used at lower concentrations, are needed for firm conclusions on a consensus signature of cI inhibition.

Besides sacc, serine was most clearly upregulated by all three cI inhibitors. This may be a consequence of the highly glycolytic state of the cells with inhibited respiration. The glycolysis intermediate 3-phosphoglycerate is a precursor in serine biosynthesis. Previous studies have already indicated that in mitochondrial disorders the elevated levels of intracellular serine correlate with increased glucose consumption [108].

**Figure 7:**
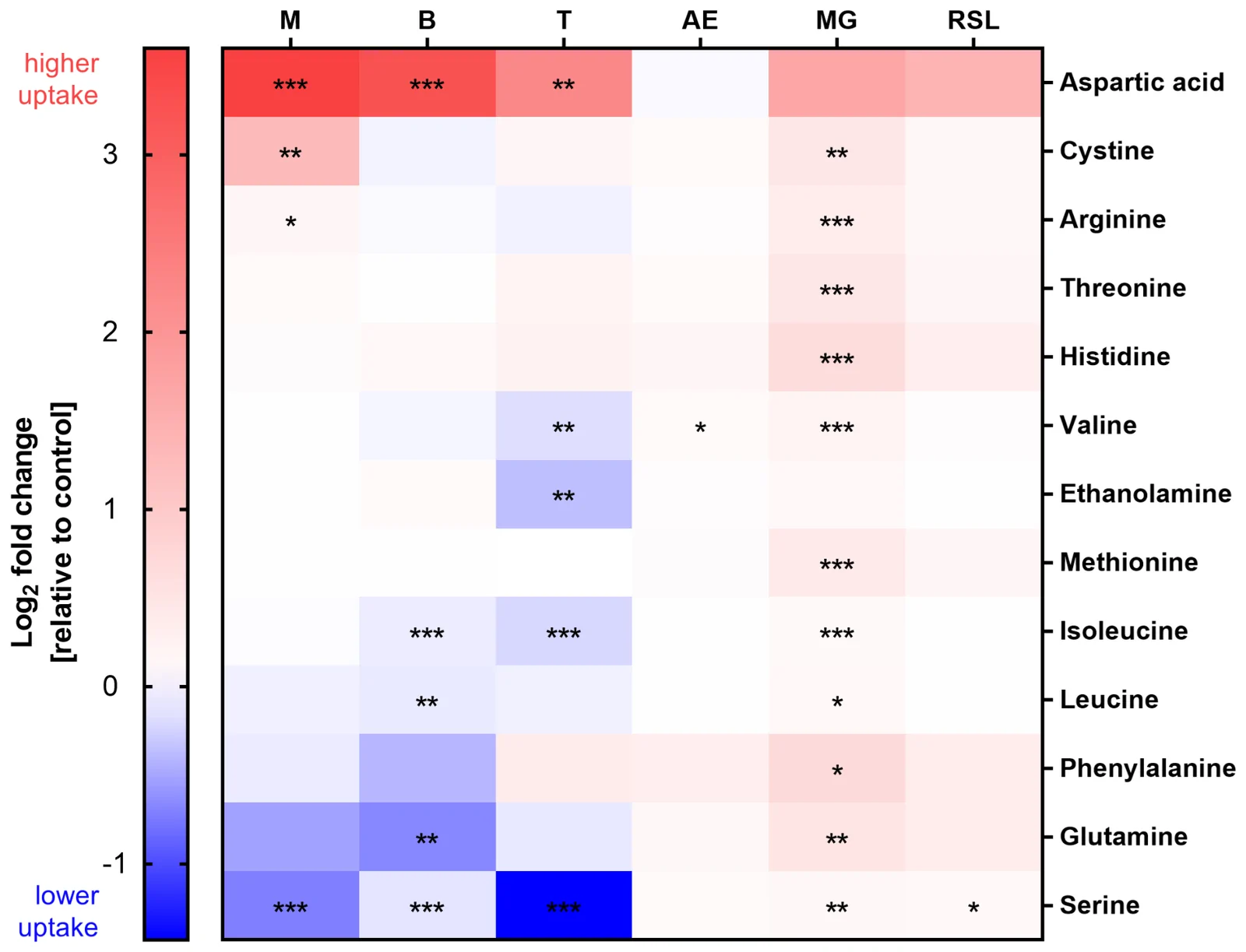
Changes in amino acid uptake rates of LUHMES cells after exposure to various toxicants. Pre-differentiated d2 LUHMES cells were seeded as in Figure 6 and treated with 10 µM berberine (B), 10 µM MPP^+^ (M), 20 µM tebufenpyrad (T), 3 µM R-aerugine (AE), 30 nM MG-132 (MG) or 30 nM RSL3 (RSL) for 24 h, before the amino acid composition of the medium was analyzed. Following the definition used in Figure 2, we considered concentration losses in the medium as “cellular uptake”. All data are normalized to the uptake rates for the respective amino acid in solvent-treated cells, and they are expressed as log2 fold changes. The heat map displays all amino acid uptake rates that were changed significantly by at least one of the toxicants. Negative values (blue) show a decrease in uptake rate (i.e., less depletion from the medium was measured); positive values (red) indicate an increased uptake (i.e., the depletion from the medium was increased). For statistical analysis, treated samples were compared to the respective control using a mixed model ANOVA (amino acid x treatment) followed by Dunnett’s multiple comparisons post hoc test. Significance levels are indicated by asterisks (*: adjusted *p* ≤ 0.05; **: adjusted *p* ≤ 0.01; ***: adjusted *p* ≤ 0.001).

Aspartic acid was most strongly downregulated by the three cI inhibitors. In the cytosol, aspartate can be converted to malate under the consumption of NADH. This cytosolic reaction may actually be used by the cells to counteract reductive stress caused by NADH overload [39]. Indeed, an increase in malate was earlier seen after cI inhibition [38]. The reverse reaction (malate to aspartate) is usually running in the mitochondria, and it requires NAD^+^ as a cofactor [109]. Sullivan et al. also showed that the loss of mitochondrial respiratory chain function and the thereby caused lack of electron acceptors (NAD^+^) leads to an impairment of aspartate biosynthesis. In line with this, we observed that cI inhibitors strongly and consistently decreased aspartate levels (Figure 6), even though cells tried to meet their need by increased uptake from the medium (Figure 7). Notably, Sullivan and colleagues showed that supplementation of aspartate was sufficient to restore the proliferation capacity of cells with inhibited respiration [86].

### 3.6. Overarching Biochemical Themes of Amino Acid Changes Triggered by cI Inhibitors

We were interested in whether the changes in various amino acids may be explained by some overarching biochemical theme. For instance, it has been suggested [49] that inhibition of mitochondria might trigger an increased utilization of amino acids for energy metabolism. This would require a “nitrogen sink”, where cells can dispose of an excess of this element that results from the use of N-containing amino acids instead of N-less sugars and lipids.

The increased need for nitrogen removal is supported by our findings of an increased Asn/Asp ratio. The Asn/Asp ratio of 0.8 in untreated cells was raised by berberine and MPP^+^ to 1.5 and 3.0, respectively. The same trend can be seen for the Gln/Glu ratio, which was 1.0 in untreated cells and 2.4 in cells treated with MPP^+^ or berberine (Figure S11). These changes fully confirm earlier findings [38]. The changes observed for ammonia, ornithine, Ala and Arg are also in accordance with the concept of nitrogen removal by cells (Figure 6 and Figure S11).

The effects triggered by tebufenpyrad did not follow the above trend. One potential explanation is that cells were functionally impaired to a greater extent by this compound, so that a larger variety of biochemical processes was disrupted.

### 3.7. Comparison of Amino Acid Changes Caused by cI Inhibition and Other Mechanisms

To contrast the pattern of amino acid changes triggered by berberine and MPP^+^ with that of other stressors, we also studied three non-mitochondrial toxicants: MG-132 (proteasome inhibitor), RSL3 (ferroptosis inducer) and aerugine (oxidative stress-inducing neurotoxicant). We used for each compound the highest non-cytotoxic concentration, as determined by viability testing (Figure S12). Even at these relatively high exposure conditions, none of the compounds triggered a significant deregulation of any intracellular amino acid (Figure S13). This clearly differed from the effects of the mitotoxicants (Figure 6). Most importantly, neither sacc nor α-AAA were significantly altered by any non-mitochondrial toxicant (Figure S13).

In addition to intracellular changes, we also examined the medium levels of amino acids and how their temporal dynamics were affected by non-mitochondrial toxicants. For RSL3 and aerugine, hardly any change occurred. They did not alter the uptake of any amino acid from the medium to cells, and changes in the other direction (secretion from cells to the medium) were negligible (Figure 7, Figures S14 and S15). In view of this, the changes observed for mitotoxicants appeared even more impressive. For MG-132, some more effects were observed. The compound increased the medium levels of Glu (higher secretion from cells) and reduced the secretion of Pro (Figures S14 and S15). It was particularly striking that the compound increased the cellular uptake of virtually all amino acids (Figure 7). A mechanistic explanation for this is that MG-132 blocked the endogenous protein turnover and therefore depleted cells of recycled amino acids. This deficit was obviously compensated for by an increased recruitment of amino acids from the medium, so that the overall cellular levels were kept constant (Figure S13). Thus, amino acid changes triggered by some non-mitochondrial toxicants appear also to follow an overarching biochemical theme. However, there were clear differences in pattern between the mitotoxicants studied here and MG-132. These data suggest that the strong changes triggered by cI-inhibitors are relatively specific. This needs additional experimental verification, but the finding is consistent with the concept that reductive stress (typical consequence of cI inhibition) does not only disturb a random pattern of metabolites, but rather causes a coordinated metabolic reprogramming [39].

In summary, the pattern of changes triggered by the two specific cI inhibitors studied here (MPP+ and berberine) is characterized by distinct changes in intracellular amino acid levels, driven by the need of cells to dispose of excess nitrogen and by reductive stress. This is accompanied by an intensive exchange (uptake and release) with the extracellular medium. This comprehensive overview of amino acid changes indicated that Lys itself was neither changed concerning its cellular concentrations, nor concerning its exchange with the extracellular medium (see also Supplementary Discussion in the Supplementary Information PDF file). The processes triggered by cI inhibition clearly differed from those observed for a panel of diverse non-mitochondrial toxicants.

### 3.8. Time- and Concentration-Dependent Changes in Sacc and α-Aminoadipic Acid

After having given the overall amino acid changes due consideration, we refocused the final studies on the Lys catabolites sacc and α-AAA. One obvious question was whether these compounds could pass neuronal cell membranes. To avoid dilution phenomena due to transport from cells to the medium, we used here a reverse experimental design: LUHMES neurons were exposed for 24 h to the two compounds (individually), and then their intracellular levels were measured. At the concentrations used here (30 µM sacc; 30 µM α-AAA), no toxicity was observed (Figure S16).

**Figure 8:**
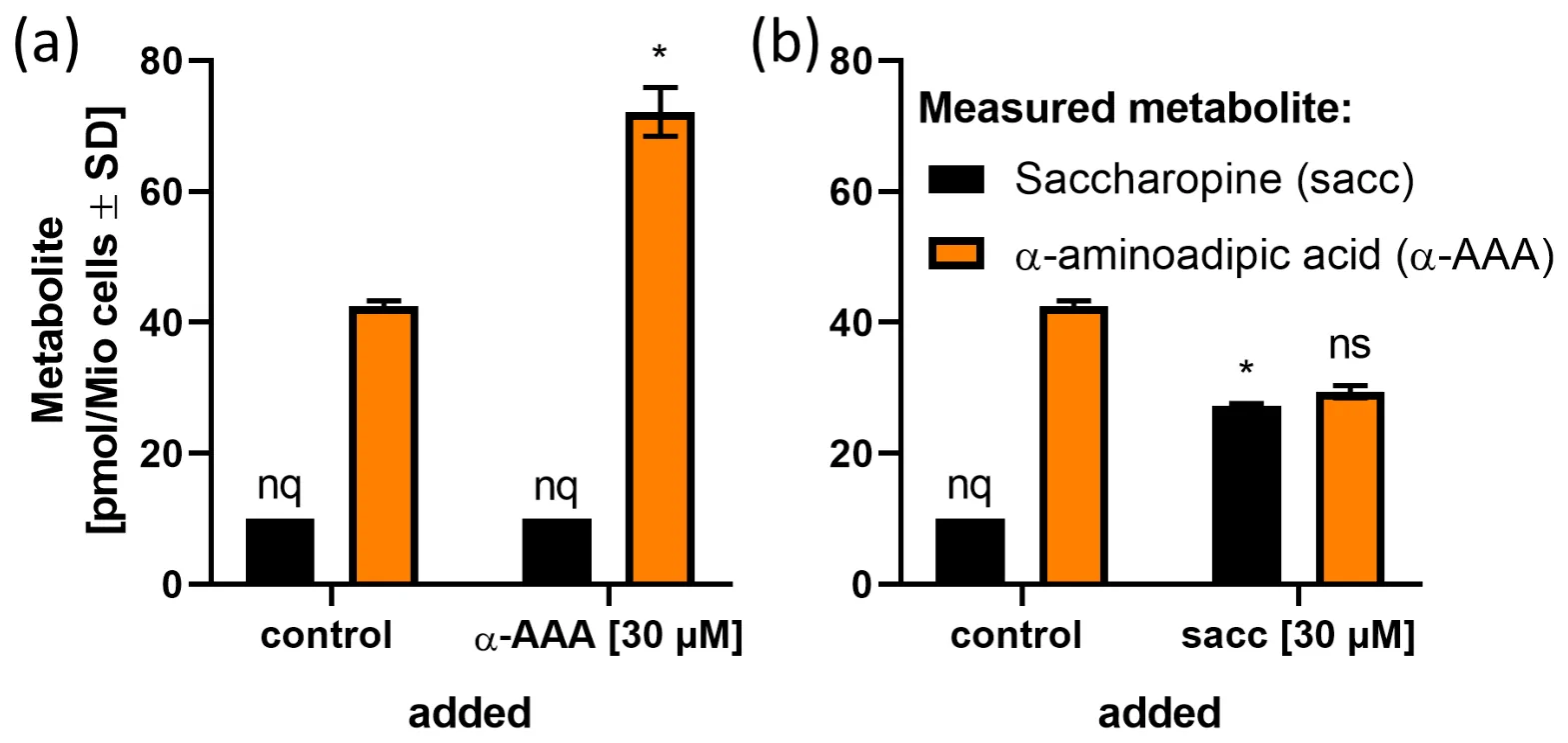
Detection of saccharopine and α-aminoadipic acid after mitochondrial inhibition. Pre-differentiated d2 LUHMES cells were seeded as in Figure 6. Cells were treated with 30 µM α-aminoadipic acid (**a**) or 30 µM saccharopine (**b**). Saccharopine (sacc) and α-aminoadipic acid (α-AAA) were quantified. Data are expressed as the average ± SD of 2 independently prepared samples (note the low variation in data from 2 different experiments). Low levels of saccharopine were detectable under the control conditions, but the level was too low to quantify (nq: not quantifiable). The height of the respective bars indicates the quantification limit of the analytical method. For statistical analysis, treated samples were compared to the respective control using a 2-way ANOVA followed by Dunnett’s multiple comparisons post hoc test. Significance levels are indicated by asterisks (*: *p* ≤ 0.05; ns: not significant).

**Figure 9:**
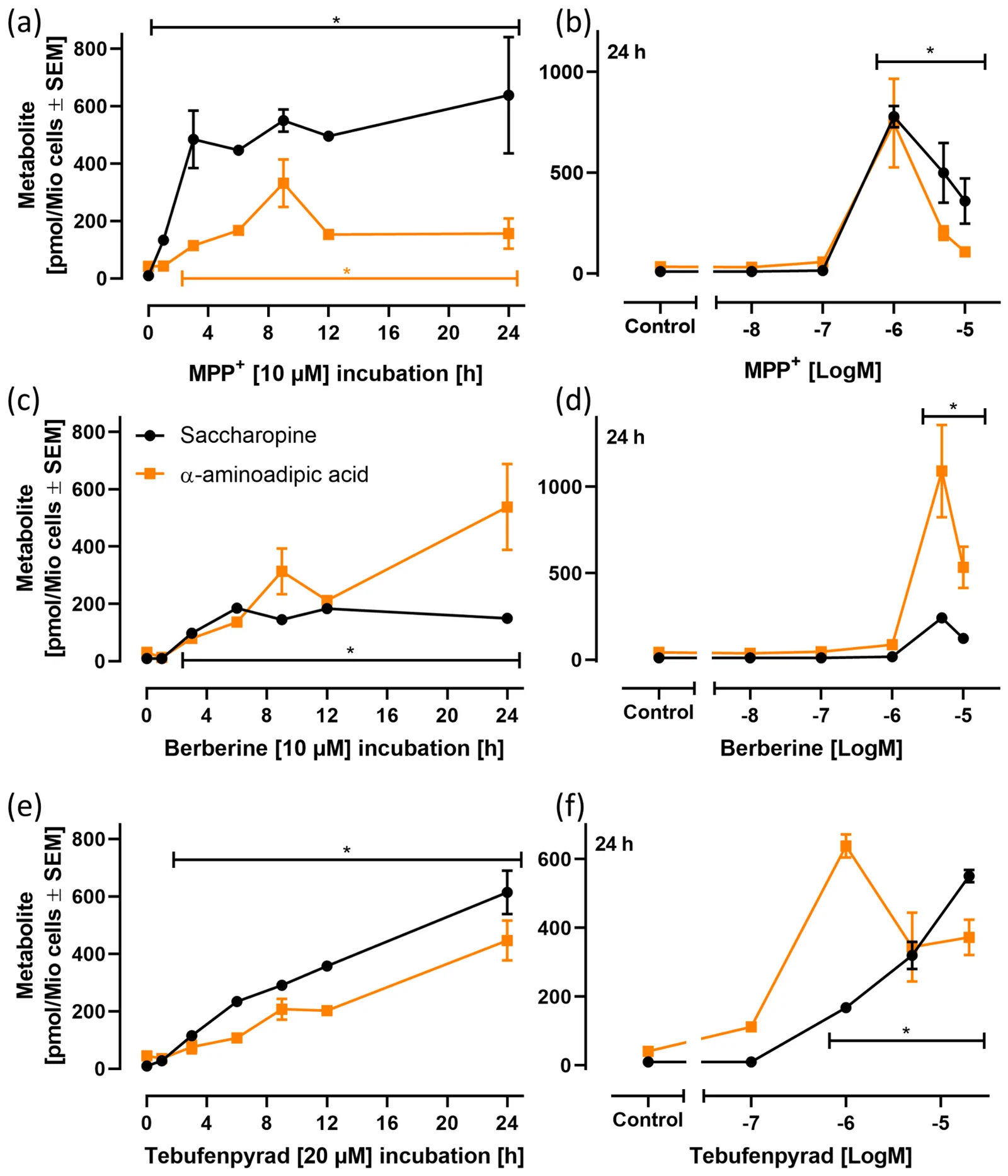
Time- and concentration-dependent accumulation of saccharopine and α-aminoadipic acid after cI inhibition. Pre-differentiated d2 LUHMES cells were seeded as in Figure 6. For time-course experiments (**a**,**c**,**e**), cells were treated with 10 µM berberine (**a**), 20 µM tebufenpyrad (**c**) or 10 µM MPP^+^ (**e**) for 1 to 24 h. Serial dilutions of the test compounds (**b**,**d**,**f**) were incubated for 24 h. The highest concentration was used in the respective time-course experiment. Saccharopine and α-aminoadipic acid were quantified. Data are expressed as the average ± SEM of 3 to 4 independently prepared samples. For statistical analysis, treated samples were compared to the respective control using a 2-way ANOVA followed by Dunnett’s multiple comparisons post hoc test. Significance levels are indicated by asterisks (*: *p* ≤ 0.05).

In this experiment, sacc was below the quantification limit (LOQ) in untreated cells (<10 pmol/10^6^ cells). Note that in the general amino acid analysis (Figure 6), sacc was slightly higher (12 µM), so that fold changes could be calculated. In cases of sacc levels below the LOQ, we suggest using a conservative fold change estimate (assuming a cellular content of 10 µM), if desired. The most exact way to indicate the obtained data is to give the absolute levels for each condition. We chose this option in Figure 8 and Figure 9. The metabolite α-AAA could be reliably detected and quantified (43 ± 0.8 pmol/10^6^ cells). Exposure of cells to external α-AAA led to a significant increase in the intracellular α-AAA content without affecting the intracellular sacc content. Conversely, sacc treatment increased its intracellular content to 27 ± 0.4 pmol/10^6^ cells, without affecting the intracellular α-AAA content. These results confirm that both compounds may cross plasma membranes to some degree, and that our methods (AAQ, AAQ2) are suitable to quantify intracellular sacc and α-AAA, as well as changes in their levels (Figure 8). Future studies may address the mechanistic basis of membrane transport and possibly examine subcellular distribution.

In a final set of experiments, we investigated the time- and concentration-dependent increase in sacc and α-AAA concentrations due to exposure to the three cI inhibitors (berberine, tebufenpyrad, MPP+). Our study on concentration-response relationships is at present preliminary and goes beyond the core statement of the manuscript. However, the data indicate the range of toxicant concentrations relevant for the increase in sacc. This correlates very well with the concentration range at which mitochondria are impaired (as measured earlier), and neurite growth is inhibited [21,38,105].

We found a significant “up-regulation” of the metabolites already at 3 h after exposure to the inhibitors (Figure 9). In the case of MPP^+^, sacc was already increased after 1 h, and its level reached a plateau after 3 h (520 pmol/10^6^ cells; corresponding to an intracellular concentration of 350 µM). The levels of α-AAA plateaued after 6 h at 160 pmol/10^6^ cells (corresponding to 107 µM). This indicates that sacc and α-AAA not only qualify as potential biomarkers of damage, but they are early indicators of cI inhibition that change long before any evident cytotoxicity can be observed. The response to MPP^+^ was interesting with respect to our observation that α-AAA failed to be up-regulated in the 24 h experiment (see Figure 6). The detailed time course data show that α-AAA is clearly up-regulated at earlier time points. Moreover, the concentration response data show a peak at relatively low concentrations (as for berberine). At exposure to 1 µM MPP^+^, there was a clear up-regulation, comparable in extent to that of the other inhibitors.

While all mitotoxicants triggered an up-regulation of sacc and α-AAA, there seemed to be differences in the individual responses. Berberine triggered a more or less continuous increase in cellular concentrations for 12 h. After 24 h, α-AAA appeared to be more abundant than sacc. The concentration-response was very sharp. Berberine concentrations of ≥5 µM were required to induce a significant increase in both metabolites (while 1 µM was not sufficient). An increase of berberine to 10 µM led to a lesser, but still significant accumulation of sacc and α-AAA. Follow-up work on such responses at earlier time points will be interesting (Figure 9c,d).

Tebufenpyrad led to a steady increase in sacc and α-AAA over the entire 24 h time window, with sacc regulated to a similar extent as α-AAA. Tebufenpyrad concentrations ≥ 1 µM (far below the cytotoxicity threshold but clearly blocking cI) already led to a profound increase in the metabolites. The levels of α-AAA had a steeper concentration-dependence than those of sacc (Figure 9e,f). The change in the α-AAA/sacc-ratio with increasing tebufenpyrad concentrations may indicate a difference in underlying mechanisms of action triggered by increasing concentrations of the toxicant (exceeding the levels required for cI inhibition).

To provide an outlook for the present study, we plan to study the effects of inhibition of other mitochondrial targets. We analyzed here, in a first pilot experiment, the changes in intracellular sacc and α-AAA triggered by antimycin A, a cIII inhibitor. Sacc was significantly increased by antimycin A treatment (23 pmol/10^6^ cells), whilst α-AAA levels were not affected (Figure S17). This pattern resembled that of 10 µM MPP^+^. It is possible that antimycin A would also lead to α-AAA under different conditions (as shown in Figure 9 for MPP+). The preliminary data on antimycin A suggests that sacc is a general indicator of mitochondrial dysfunction, but more extensive data on this will need to be generated with a wide panel of mitochondrial inhibitors.

## 4. Conclusions

**Figure 10:**
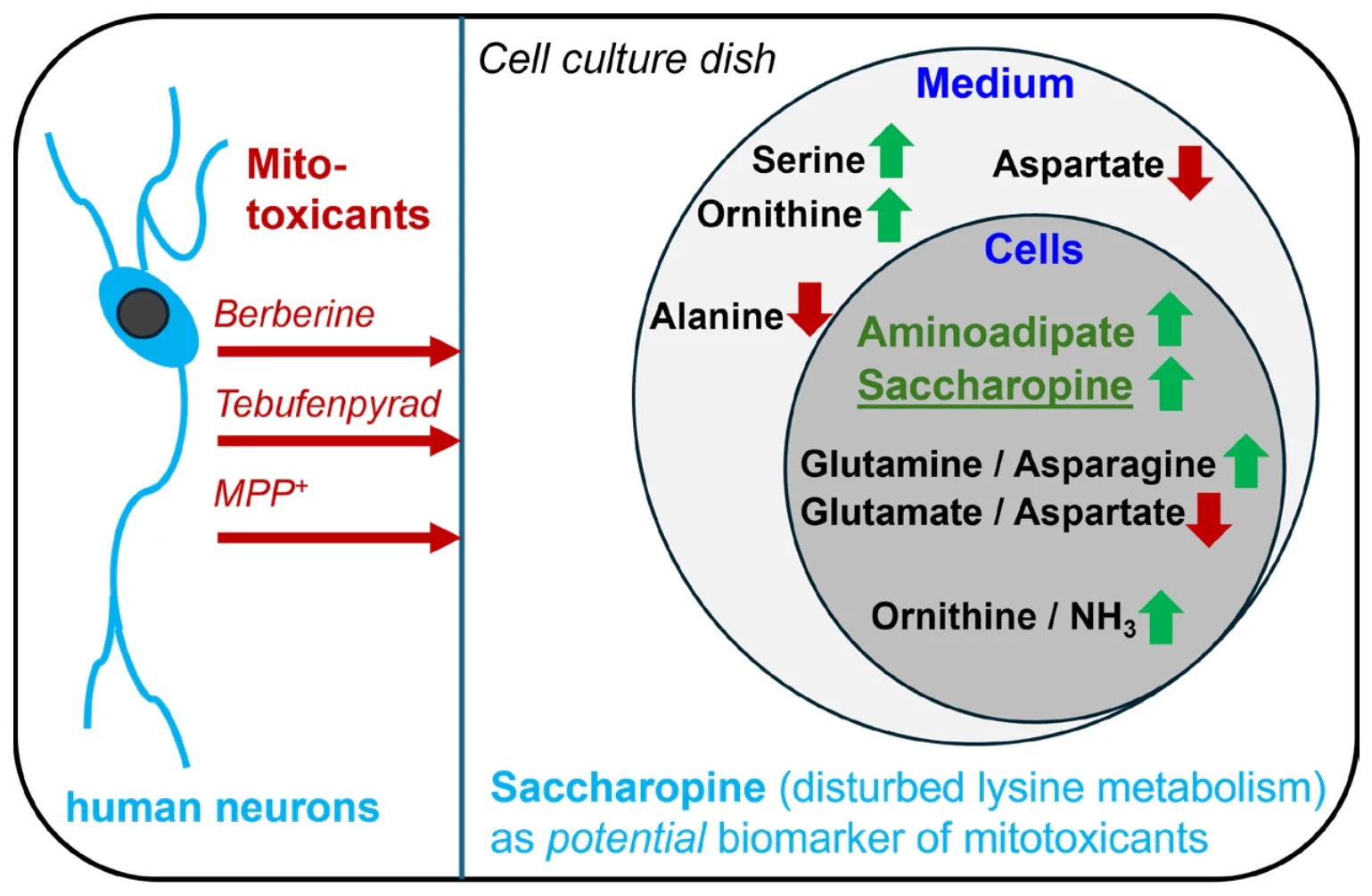
Overview of overarching study outcomes. Neuronal cultures (differentiated LUHMES cells) were exposed to the three mitochondrial toxicants (all inhibitors of the mitochondrial respiratory chain complex I), and also to three non-mitochondrial toxicants (not visualized). Then, amino acid levels (proteinogenic amino acids and several others) were analyzed in cells and in the surrounding medium. Using information on the cell volume, amino acid concentrations in cells were determined before and after exposure to toxicants. The changes occurring after exposure to mitotoxicants, but not after other toxic challenges to the neurons, are highlighted. Green (up) and red (down) arrows indicate the direction of regulation of metabolite levels by exposure to toxicants.

We provided here a comprehensive overview of absolute amino acid levels in LUHMES neurons and their surrounding medium. Moreover, changes triggered by six toxicants were quantified, with a particular focus on the pattern related to electron transport chain cI inhibition. The Lys catabolites sacc and α-AAA were found to be rapidly and concentration-dependently increased by three mitotoxicants, but not by non-mitochondrial toxicants (Figure 10). We suggest on this basis that they be further explored as potential biomarkers of cI inhibition and the associated reductive stress. Here, several implications of these findings, together with shortcomings and potential pitfalls, are briefly outlined:

First, we would like to indicate that this study used only one particular model system (LUHMES neurons), and that data on amino acid changes have only been generated for small sets of mitotoxicants and non-mitochondrial toxicants. This means that care needs to be taken with a generalization of the study results to other neurons (or non-neuronal cell types) and to other mitotoxicants. Further studies will be required to allow firm conclusions on these issues.

Second, the resource we provide here on cellular amino acid concentrations may have broad applicability. While there is a large number of studies that determined relative changes in metabolites in various body fluids, cells and tissues, there is an astonishing dearth of absolute quantifications of metabolite pools. Concerning cell cultures, this is partly explained by the difficulty of measuring cell volume. In addition, running the necessary calibration samples requires considerable resources to keep absolute variability between runs (samples) sufficiently low. For this reason, we used the AAQ, a particularly robust method (low coefficient of variation between runs). This traditional biochemical approach (Figure S2), proven to deliver reliable clinical data, is used nowadays rarely for cell-based studies. The need for large numbers of cells, due to the low abundance of amino acids close to the detection limit, together with the relatively long analysis time, was not a significant limitation in our study.

Second, the analytical method was important here, as changes in metabolites are often relatively minor compared to the expression levels of genes (mRNA levels) or the amounts of certain protein species. The latter may change by up to 2–3 orders of magnitude, while significant and often important metabolite (amino acid) changes are often far less than 2-fold. Our quantification approach (with low coefficients of variation between experiments) allowed us to identify relatively small, but significant, changes in the pool sizes of amino acids. Such data were not only obtained in cells, but also in the surrounding medium. The information on assumed fluxes into cells or out of cells may be useful for planning and interpreting cell culture experiments. In addition, future experiments may explore changes in sacc and α-AAA in the extracellular space of tissues (e.g., in cerebrospinal fluid) as putative biomarkers. This would require that extracellular levels of, e.g., sacc reflect intracellular increases. Testing this assumption was beyond the scope and possibilities of the current study approach, but our experimental data from cell cultures provide a rationale for follow-up studies. Even if the Lys metabolites would turn out not to be useful as CSF biomarkers, one may, alternatively, look for other signatures described here, such as a depletion of Asp. Particularly striking differences between cI inhibitors and other toxicants were also observed for Ser: the uptake from medium was decreased by mitotoxicants (as cells produced more Ser themselves). For non-mitochondrial toxicants, the reverse effect was observed (increased cellular uptake). Another biomarker pattern that may be explored is the one related to excessive ammonia production and the neuronal need to dispose of excess nitrogen.

Third, the absolute molar concentrations of sacc and α-AAA deserve some consideration. Using the cell volumes to calculate concentrations, we found that sacc (<10 µM in control cells) reached levels above 400 µM (with 600 pmol/10^6^ cells corresponding to 400 µM) after exposure to tebufenpyrad and MPP^+^, and 200 µM in the case of berberine. In our cell uptake experiment, we used a comparatively low extracellular concentration of 30 µM. This suggests that the Lys metabolites can (to some extent) cross cell membranes. Thus, they should also be able to ‘move’ out of cells into the medium (or tissue extracellular fluid). We were not able to measure the metabolites outside cells. This is explained by the dilution factor in the medium (about 10,000-fold, compared to the cell volume). This situation may be different in CSF, where the extracellular volume is low compared to the cellular mass. Similar considerations apply to α-AAA. Its cellular concentrations also reached levels of 200–400 µM (with 300 pmol/10^6^ cells corresponding to 200 µM).

Fourth, it may be astonishing that catabolites of Lys took such a prominent role here, although the parent amino acid has not been studied intensively for its disease association. Studies on Lys degradation pathways have identified rare genetic defects in humans (inborn errors of metabolism) that are strongly associated with pathologies [110,111,112,113]. Hyperlysinemia (especially type II) is known as a human disease. It is characterized by increased Lys and sometimes sacc levels. The increases in sacc arise from mutations in the gene responsible for the reduction in sacc (AASS—see Figure S9). Further diseases associated with the Lys degradation pathway are pyridoxine-dependent epilepsy disease (ALDH7A1 mutation), 2-aminoadipic and 2-oxoadipic aciduria (DHTKD1 mutation), glutaric aciduria type 1 (GCDH mutation) and glutaric aciduria type 3 (GA3 mutation). All are associated with neurological disabilities [74,82].

To interpret the relation between Lys and its metabolites, a short summary of knowledge on the respective catabolism appears important. Two pathways are known: the “pipecolic acid pathway” (localized in mitochondria, peroxisomes and the cytosol) and the “sacc pathway” (mainly mitochondria-located). Recent studies suggest that the sacc pathway is predominantly used in the brain [74,75]. In the future, one may also explore in cell culture experiments whether Lys hyper-supplementation of neuronal cultures further increases sacc production under mitochondrial stress, and whether this may have consequences on the “cell-health state”. However, already now, our study clearly identified and quantified sacc (and possibly α-AAA) as mechanistically anchored, potential biomarkers of mitotoxicity. The biochemical basis linking sacc to cI inhibition and reductive stress is that eight net reduction equivalents (in the form of 3 NADH and 1 FADH_2_) are generated in the sacc pathway (Figures S9 and S10). In situations of reductive stress, the associated biochemical reactions (downstream of sacc) can hardly proceed, and therefore sacc accumulates.

Fifth, it is at present unclear whether the increase in sacc/α-AAA measured here is only a “neutral biomarker” of metabolic changes, or whether these metabolites also contribute to pathological effects. A role of the two Lys degradation metabolites as biomarkers is supported by observations of saccharopinuria and α-aminoadipic aciduria in human disease [74,82]. Sacc also accumulates in the kidney during injury [81], but more work and evidence is required from human studies. Notably, there are also some data which suggest that the accumulation of sacc is linked to mitochondrial dysfunction in vivo [79]. The sacc pathway intermediates sacc and α-AAA have been identified to be associated with inflammation in several metabolomics studies [76,77,78]. Few studies suggest that sacc itself is a mitochondrial toxicant [79,80], and that α-AAA is potentially neurotoxic [75]. For instance, disruption of the sacc pathway in *Caenorhabditis elegans* or in mice has been shown to lead to an accumulation of sacc. This was associated with disrupted mitochondrial dynamics and subsequent mitochondrial damage [79].

Sixth, it is worth considering how the metabolic changes described here relate to other biomarkers of cell dysfunction and cell death. Many of the most established markers refer to late phases of cell death. They measure the release of cellular proteins (e.g., lactate dehydrogenase or various tissue-specific enzymes) into the environment. For apoptosis, proteolytic activity (caspase activity or cleavage of caspase substrates) may be detected long before membrane rupture. We identified two studies that examined amino acid changes when apoptosis was triggered in tumour cells. Very early upregulations of, e.g., Asp, Ala and Glu were observed, but the work was not followed up for healthy human cells [114,115]. The pattern largely differs from the cI pattern observed here (all above amino acids were down-regulated in our study). There are also studies on metabolites released from apoptotic cells. They included nucleotides, spermidine and glycolysis intermediates, but the released metabolites were explicitly labelled as “farewell signal” (occurring after cell death) [116]. Concerning ferroptosis, we included the ferroptosis inducer RSL3 here, and it altered the amino acid pattern very little. A new form of cell death, particularly important for neurons, is SARM1-dependent axonal degeneration (sarmoptosis) [117]. Its key biochemical feature is depletion of NAD^+^. This may mimic some aspects of reductive stress, and mitochondrial dysfunction may play an important role [118]. It is therefore of high interest to see whether SARM1-dependent neuronal death, e.g., triggered by vacor [119], may lead to amino acid changes and possibly sacc accumulation similar to mitotoxicants. Finally, mitochondrial dysfunction may play a role in chronic pain, e.g., in chemotherapy-induced peripheral neuropathy or in diabetic neuropathy. Several treatment options have been suggested, and the interpretation of clinical trial outcomes would benefit from a biomarker that indicates that mitochondrial dysfunction is reduced by treatments.

Seventh, we included the cI inhibitor tebufenpyrad in our study, but we had to exclude its effects from the definition of a putative pattern of amino acid changes associated with the tested complex I inhibitors. As discussed in the respective results section, we now believe that the concentration choice was not optimal. For the other cI inhibitors, the concentration chosen (based on neurite effects) was optimal to provide significant (80%) mitochondrial inhibition, but it was sufficiently low not to non-specifically affect a large number of other targets. For tebufenpyrad, the concentration affecting neurites was much higher than the concentration (EC80) inhibiting cI. We have to assume that this high concentration affected many other metabolic processes. Therefore, tebufenpyrad induced some unique amino acid changes not seen with berberine and MPP^+^: a strong up-regulation of GABA and a significant up-regulation of Pro in combination with a significant downregulation of cystathionine, taurine, Asn, Gln, Val and Thr. Interestingly, sacc and α-AAA levels were significantly (to a similar extent as with other cI inhibitors) increased in cells treated with tebufenpyrad. This suggests that sacc/α-AAA may be useful as biomarkers of cI inhibition, even when this is combined with other functional impairments. The biomarker function was also maintained at high stress levels (close to the threshold of cell death induction). This feature may be particularly important under conditions of mixture exposure, or exposure to toxicants that simultaneously affect several targets, or when considering neurodegenerative pathologies, where mitochondrial failure is accompanied by other cell pathologies (e.g., protein aggregation and disturbed proteostasis) [120,121]. In this context, our finding that none of the non-mitochondrial toxicants increased the levels of sacc/α-AAA suggests that even high levels of “unspecific” functional impairment do not change these biomarkers. Finally, an interesting observation of the concentration-response studies suggests that tebufenpyrad will trigger increases in sacc/α-AAA at sub-micromolar concentrations (where it is specific for cI). These findings are being followed up in greater detail and for a broader panel of mitochondrial inhibitors, alone and in mixtures.

Eighth, the availability of mechanism-based biomarkers, such as sacc, may help address one of the key problems of neurotoxicology: how can one relate findings in short-term studies to chronic outcomes in humans after decades of exposure [26,122,123,124]. Countless studies in model systems (cells and animals) have shown that severe inhibition of mitochondria (e.g., by MPP^+^ or tebufenpyrad) can lead to neuronal death (in particular death of dopaminergic neurons, relevant to Parkinson’s disease). With the exception of MPP^+^ (in some accidental poisoning situations of drug addicts), environmental agents do not cause such immediate neuronal death in humans. However, there is strong epidemiological evidence that some mitotoxicants (e.g., rotenone) lead to dopaminergic neurodegeneration with parkinsonian motor deficits after chronic exposure [27,125,126,127,128]. There is little information on what happens during the non-symptomatic exposure time, and how non-lethal events occurring over long time periods are linked to the final cell demise. What is needed to understand causal links between exposure to mitotoxicants and neurodegeneration is the identification of mechanisms of sublethal stress (that may, after chronic exposure, lead to neuronal cell death) and of respective mechanism-linked biomarkers. An elevation of sacc above background levels may be explored as a biomarker of mild, but still biochemically effective mitochondrial stress. Availability of such measures would help to convert descriptive adverse outcome pathways to mathematical models that quantitatively connect disease outcomes with mild mitochondrial inhibition over prolonged time periods.

## Figures and Tables

**Table 1:** Uptake of amino acids from cell culture medium ^1^.

Amino Acid	% Internalized by Cells
Aspartate	1.1 ± 1.0
Threonine	4.7 ± 0.6
Serine	18.4 ± 0.7
Glutamine	13.6 ± 1.2
Valine	14.2 ± 0.5
Cystine	8.6 ± 1.4
Methionine	8.4 ± 0.7
Isoleucine	22.6 ± 0.4
Leucine	23.4 ± 0.8
Tyrosine	3.8 ± 1.4
Phenylalanine	4.6 ± 1.6
Histidine	4.2 ± 0.8
Lysine	5.4 ± 0.7
Ethanolamine	35.5 ± 3.1
Arginine	6.6 ± 0.5

^1^ LUHMES cells were plated on d2 into 10 cm dishes and incubated for 24 h at 37 °C and 5% CO_2_, before the amino acid composition of the medium was analyzed. As a negative control, differentiation medium was incubated without cells. The amino acid content after incubation with cells was subtracted from the negative control and is expressed as % ± SD of the negative control to indicate the uptake of individual amino acids from the medium. See more data in Figure 2.

## Data Availability

All raw data are compiled as Supplementary Material (Excel format). The file is also available in Zenodo [63]. Additional data are also provided in the Supplementary Information file (PDF format). All other relevant data and information will be made freely available upon request to the corresponding author.

## Supplementary Materials

The following supporting information can be downloaded at https://www.mdpi.com/article/10.3390/cells15191804/s1, SupplementaryData.xlsx; Supplementary discussion; Figure S1: Post-column derivatisation reaction of amino acids with ninhydrin; Figure S2: Method used for the separation of amino acids; Figure S3: Intracellular amino acid concentrations of LUHMES cells; Figure S4: Chromatogram of the amino acid standard including saccharopine (AAQ); Figure S5: Chromatogram of the amino acid standard highlighting the common peak of cysteine and α-aminoadipic acid with the standard method (AAQ); Figure S6: Method used for the separation of cysteine and α-aminoadipic acid (AAQ2); Figure S7: Metabolites detected by AAQ and AAQ2; Figure S8: Specific accumulation of the L-lysine degradation metabolites saccharopine and α-aminoadipic acid upon mitochondrial stress; Figure S9: Overview of the L-lysine degradation pathway and the expression of related genes enzymes in LUHMES cells; Figure S10: The role of the cellular redox state in controlling the saccharopine pathway; Figure S11: cI inhibitors affect high “N”-content and “N”-acceptor amino acids; Figure S12: Assessment of viability and neurite outgrowth in the presence of various toxicants; Figure S13: Toxicant-induced changes of intracellular amino acids and related compounds by aerugine, MG-132 and RSL3; Figure S14: Amino acid uptake and secretion rates of LUHMES cells after treatment with various toxicants; Figure S15: Changes in amino acid secretion rates of LUHMES cells after exposure to various toxicants; Figure S16: Assessment of viability and neurite outgrowth in the presence of L-lysine degradation products; Figure S17: cIII-inhibition by Antimycin A increases the intracellular saccharopine content; Supplementary references.

## Abbreviations

The following abbreviations are used in this manuscript:

α-AAA	α-aminoadipic acid
AAQ	Amino acid quantification; method to quantify most amino acids and related metabolites
AAQ2	Amino acid quantification 2; method to quantify α-aminoadipic acid and cysteine
AE	Aerugine
AntiA	Antimycin A
AO	Adverse outcome
AOP	Adverse outcome pathway
Asn	Asparagine
Asp	Aspartate
B	Berberine
BCAA	Branched-chain amino acid
BMC25	Benchmark concentration leading to a 25% effect
BPA	Bisphenol A
cI	Complex one of the mitochondrial electron transport chain
cIII	Complex three of the mitochondrial electron transport chain
CSF	Cerebrospinal fluid
CoA	Coenzyme A
db-cAMP	Dibutyryl cyclic adenosine monophosphate
DMEM/F12	Dulbecco’s Modified Eagle Medium: Nutrient Mixture F12
DMSO	Dimethyl sulfoxide
dx	Day of differentiation x
FAD	Fully oxidized flavin adenine dinucleotide
FADH_2_	Fully reduced flavin adenine dinucleotide
FGF-2	Fibroblast growth factor 2
GDNF GSH	Glial cell line-derived neurotrophic factor Glutathione
KE(s)	Key event(s)
M	MPP^+^
MG	MG-132
MIE	Molecular initiating event
MPP^+^	1-methyl-4-phenylpyridinium
MPTP	1-methyl-4-phenyl-1,2,3,6-tetrahydropyridine
NA	Neurite area
NAD^+^	Oxidized nicotinamide adenine dinucleotide
NADH	Reduced nicotinamide adenine dinucleotide
NADP^+^	Oxidized nicotinamide adenine dinucleotide phosphate
NADP’H	Reduced nicotinamide adenine dinucleotide phosphate
PBS	Phosphate-buffered saline
PLO	Poly-L-ornithine
RSL	RSL3
sacc	Saccharopine
T	Tebufenpyrad
V	Viability
