# Supplementary material for: Saccharopine and α-Aminoadipic Acid as Potential Biomarkers for Mitochondrial Stress in Neurons
Source: Cells. 2026 Oct 2;15(19):1804. doi: 10.3390/cells15191804 (PMC13664059; doi:10.3390/cells15191804)
Supplement: Supplementary file 1 [file cells-15-01804-s001.zip › cells-4583792-supplementary.pdf]

Saccharopine and α-aminoadipic acid as potential biomarkers for mitochondrial stress in neurons.

Anna-Katharina Ückert[1,], Anja Land[1,*], Regina Pape[1], Mahshid Alimohammadi[1], Ivano Amelio[2], Marcel Leist[1]*

Table of Contents

Information	Page (P)	Short description / title
Text	P 2	Supplementary discussion
Fig. S1	P5	Post-column derivatisation reaction of amino acids with ninhydrin.
Fig. S2	P6	Method used for the separation of amino acids.
Fig. S3	P7	Intracellular amino acid concentrations of LUHMES cells.
Fig. S4	P7	Chromatogram of the amino acid standard including saccharopine (AAQ).
Fig. S5	P8	Chromatogram of the amino acid standard highlighting the common peak of cysteine and α-aminoadipic acid with the standard method (AAQ).
Fig. S6	P8	Method used for the separation of cysteine and α-aminoadipic acid (AAQ2).
Fig. S7	P9	Metabolites detected by AAQ and AAQ2
Fig. S8	P10	Specific accumulation of the L-lysine degradation metabolites saccharopine and α-aminoadipic acid upon mitochondrial stress.
Fig. S9	P11	Overview of the L-lysine degradation pathway and the expression of related genes enzymes in LUHMES cells.
Fig. S10	P13	The role of the cellular redox state in controlling the saccharopine pathway.
Fig. S11	P14	cI inhibitors affect high “N”-content and “N”-acceptor amino acids
Fig. S12	P15	Assessment of viability and neurite outgrowth in the presence of various toxicants.
Fig. S13	P16	Toxicant-induced changes of intracellular amino acids and related compounds by aerugine, MG-132 and RSL3
Fig. S14	P18	Amino acid uptake and secretion rates of LUHMES cells after treatment with various toxicants.
Fig. S15	P20	Changes in amino acid secretion rates of LUHMES cells after exposure to various toxicants.
Fig. S16	P21	Assessment of viability and neurite outgrowth in the presence of L-lysine degradation products.
Fig. S17	P22	cIII-inhibition by Antimycin A increases the intracellular saccharopine content
Text /Ref	P23	Supplementary references

Supplementary discussion

1 General examples of amino acid neurotoxicity

Although amino acids are essential elements of cellular metabolism, they have also been linked to several human neurotoxicities, when they are present at excessive amounts or in wrong locations. One reason is that some amino acids, like glutamate, not only participate in intracellular metabolic processes, but also have a role as intercellular messengers and triggers of receptor responses. Another reason is that the levels of amino acids determine various upstream and downstream reactions, so that other, potentially toxic intermediates accumulate or get depleted. Four well-known examples of amino acid neurotoxicity (beyond the well-known role of glutamate in excitotoxicity) are: (i) phenylketonuria; (ii) hyperglycinemia, (iii) maple syrup urine disease, and (iv) various modulations of the transsulfuration pathway.

1.1 Phenylketonuria is an autosomal recessive disorder caused by the loss of phenylalanine hydroxylase activity. It is caused by the accumulation of phenylalanine, which may lead to significant impairments to brain development, severe neurological symptoms and cognitive impairments [1-3].

1.2 Hyperglycinemia may be associated to the mitochondrial glycine cleavage system in primary, non-ketotic glycine encephalopathy. Moreover, a secondary hyperglycinemia is associated with deletions of pyrroline-5-carboxylate reductase 2 [4]. The glycine accumulation may lead to microcephaly and hypomyelination, via its agonist role on various receptors. [5].

1.3 Maple syrup urine disease: Branched-chain amino acids (BCAA) are transaminated to their respective branched-chain keto acids (BCKA): leucine to α-ketoisocaproate, valine to α-ketoisovalerate, and isoleucine to α-keto-β-methylvalerate [6]. Impairment of the further conversion of BCKA, e.g. due to defects of the branched-chain α-ketoacid dehydrogenase (BCKAD), can lead to disorders like maple syrup urine disease, which has neurological implications like encephalopathy [7]. The leucine metabolite α-ketoisocaproate was shown to be neurotoxic and to impair mitochondrial respiration causing an elevated intracellular reactive species formation [8].

1.4 The transsulfuration pathway allows an alternative supply of cysteine and is often activated under conditions of cellular stress. It allows a conversion of methionine to cysteine (and glutathione) via intermediates, like homocysteine and cystathionine. Dysfunctions have been linked to neurodegenerative syndromes, e.g. cystathioninelyase deficiency to Huntington's disease [9] or hyperhomocysteinemia to various other disorders [10,11].

2. The specific connection of lysine degradation and disease

Lysine can be degraded via the pipecolic acid pathway or the mitochondrial saccharopine pathway. The former is partially localised in mitochondria, peroxisomes and the cytosol [12]. The pipecolic acid pathway has previously been thought to be the main route of lysine degradation in the brain. Recent studies have refuted this assumption [13]. Human astrocytes, neuronal progenitor cells and also fibroblasts express the genes necessary for the saccharopine pathway [13,14]. Metabolomic flux analysis provided further evidence for lysine degradation via the saccharopine pathway. Notably, eight net reduction equivalents in the form of three NADH

and one $FADH_2$ are generated in this process (Figure S9, S10) [12]. The saccharopine pathway intermediates saccharopine and α-aminoadipic acid have previously been identified as markers for mitochondrial toxicity [12,15]. They have been identified to be associated with inflammation in several metabolomics studies [16-18]. Saccharopine is a reported mitochondrial toxicant [19,20], and α-AAA has been reported as potentially neurotoxic [13]. In *C. elegans* and mice, a disruption of the saccharopine pathway leads to an accumulation of saccharopine, which results in disrupted mitochondrial dynamics and subsequent mitochondrial damage [19]. Saccharopine also accumulates in the kidney during injury [21].

Notably, hyperlysinemia as such is known as a human disease. It is characterized by increased lysine and sometimes saccharopine levels and can be caused by mutations in the gene responsible for the reduction of saccharopine (AASS). Further diseases associated with the lysine degradation pathway are pyridoxine-dependent epilepsy disease (ALDH7A1 mutation), 2-aminoadipic and 2-oxoadipic aciduria (DHTKD1 mutation), glutaric aciduria type 1 (GCDH mutation) and glutaric aciduria type 3 (GA3 mutation). All are associated with neurological disabilities [12,22].

3. The connection of mitochondrial dysfunction and amino acid changes

The increased levels of Tyr, Phe, Ile, Val, Met and Thr upon complex I inhibition are in agreement with biochemical knowledge on amino acid utilization by the tricarboxylic acid cycle (TCA). Phe and Tyr are converted to fumarate, which enters the TCA cycle. Likewise, Ile, Met, Thr and Val are converted to succinyl-CoA, another intermediate of the TCA cycle. Disturbances in the TCA cycle could lead to elevated levels of the precursor amino acids as bottleneck buildup, since their metabolites are not used up [15].

Asp has been identified as a limiting metabolite for cancer cell growth under hypoxia. Lack of sufficient oxygen supply is common in tumors and leads to electron transport chain (ETC) dysfunction. Metabolomics studies demonstrated that a reduced ETC activity leads to significantly reduced levels of Asp and consequently impaired proliferation. An increased import of Asp via SLC1A3 can counteract this [23].

While all amino acids are required for biosynthetic processes, in particular during cell proliferation or cell growth, Asp takes an exceptional role. It is not only required for protein synthesis, but also pyrimidine and AMP synthesis. Its bottleneck function for tumor growth is well known [23-25]. Asp also represents the link between mitochondrial function and cell proliferation [26,27].

4. Exemplification of a complex cell biological process being linked to amino acid patterns

For instance, T-cells switch from mitochondrial to glycolytic energy supply during activation [28]. This is accompanied by a drop of the nitrogen-rich amino acids Arg, Orn and N-Ac-Orn. Elevating L-Arg levels induced global metabolic changes including a shift from glycolysis to oxidative phosphorylation in activated T cells and it promoted the generation of central memory-like cells endowed with higher survival capacity and, in a mouse model, anti-tumor

activity. A study examining 429 metabolites during T-cell activation found that 49 increased significantly (Log2 fold change [fc] > 1; $p < 0.01$). Only 14 were decreased in activated T cells, of which three, Arg, Orn, and N-Ac-Orn, belonged to the same metabolic pathway (linked to nitrogen usage) [29].

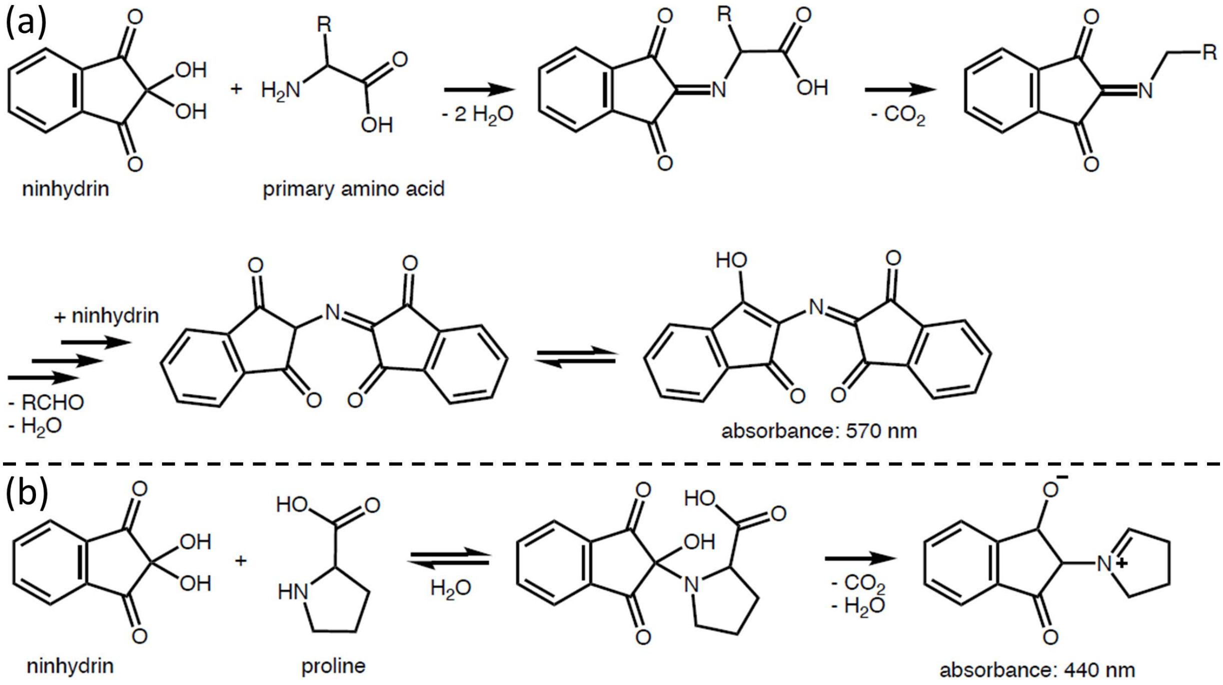

Supplementary Figure S1: Post-column derivatisation reaction of amino acids with ninhydrin.

The detection of the amino acid analyzer is based on a continuous post-column derivatisation reaction of the eluate and a subsequent absorbance analysis at 570 nm and 440 nm. The eluate is mixed post-column with ninhydrin to initiate the reaction. (**a**) Primary amino acids react with ninhydrin to a Schiff base, which is decarboxylated and then hydrolyzed to an aldehyde (leaving the reaction sequence) and various intermediates (reduced ninhydrin, hydrindantin, ammonia) that undergo a condensation reaction with a second ninhydrin molecule to form Ruhemann's purple. Ruhemann's purple has an absorbance maximum at 570 nm. (**b**) Secondary amines react with ninhydrin to form a yellow addition product with an absorbance maximum at 440 nm.

Time [min]	Type of function	Parameter of function			
Initialisation	Composition of solvent	A: 100%	B: 0%	C: 0%	D: 0%
Initialisation	Flow rate	0.45 ml/min			
Initialisation	Upper limit of pressure	120 bar			
Initialisation	Amino flow rate	0.25 ml/min			
Initialisation	Amino reactor temperature	130 °C			
Initialisation	Amino column temperature	40 °C			
Initialisation	Amino reagent valve	Ninhydrin			
1	Amino column temperature	40 °C			
2	Amino auto zero				
11.2	Composition of solvent	A: 100%	B: 0%	C: 0%	D: 0%
17.5	Composition of solvent	A: 80%	B: 20%	C: 0%	D: 0%
45	Composition of solvent	A: 79%	B: 21%	C: 0%	D: 0%
60	Composition of solvent	A: 9%	B: 91%	C: 0%	D: 0%
60	Amino column temperature	40 °C			
69	Amino column temperature	58 °C			
75	Composition of solvent	A: 0%	B: 95%	C: 5%	D: 0%
77	Composition of solvent	A: 0%	B: 45%	C: 55%	D: 0%
77.1	Composition of solvent	A: 0%	B: 0%	C: 100%	D: 0%
85	Amino column temperature	70 °C			
88	Amino column temperature	70 °C			
97	Composition of solvent	A: 0%	B: 0%	C: 98%	D: 2%
103	Composition of solvent	A: 0%	B: 0%	C: 90%	D: 10%
107	Composition of solvent	A: 0%	B: 0%	C: 82%	D: 18%
116	Amino column temperature	74 °C			
122	Amino reagent valve	Wash (25% ethanol, 25% isopropanol)			
125	Composition of solvent	A: 0%	B: 0%	C: 76%	D: 24%
125	Amino column temperature	74 °C			
125.1	Composition of solvent	A: 0%	B: 0%	C: 0%	D: 100%
129	Composition of solvent	A: 0%	B: 0%	C: 0%	D: 100%
129.1	Composition of solvent	A: 100%	B: 0%	C: 0%	D: 0%
135	Amino column temperature	40 °C			
151	Composition of solvent	A: 100%	B: 0%	C: 0%	D: 0%
300	Amino column temperature	40 °C			

Supplementary Figure S2: Method used for the separation of amino acids (AAQ).

The chromatographic method (signified here as “AAQ“) is described. The time table indicates the changes in the buffer composition and the column temperature used for the sequential elution of amino acids. It allows for the quantification of saccharopine.

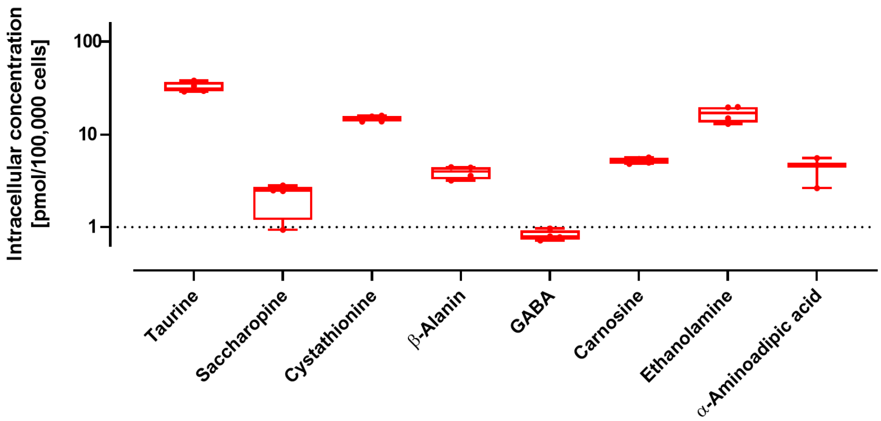

Supplementary Figure S3: Intracellular amino acid concentrations of LUHMES cells.

LUHMES cells were plated on d2 into 10 cm dishes and incubated for 24 h at 37 °C and 5% CO_2 before cells were lysed and their amino acid composition was analyzed. Data are presented as box-whisker plots with a total of four independently-prepared replicates. All data can be found in the excel supplement.

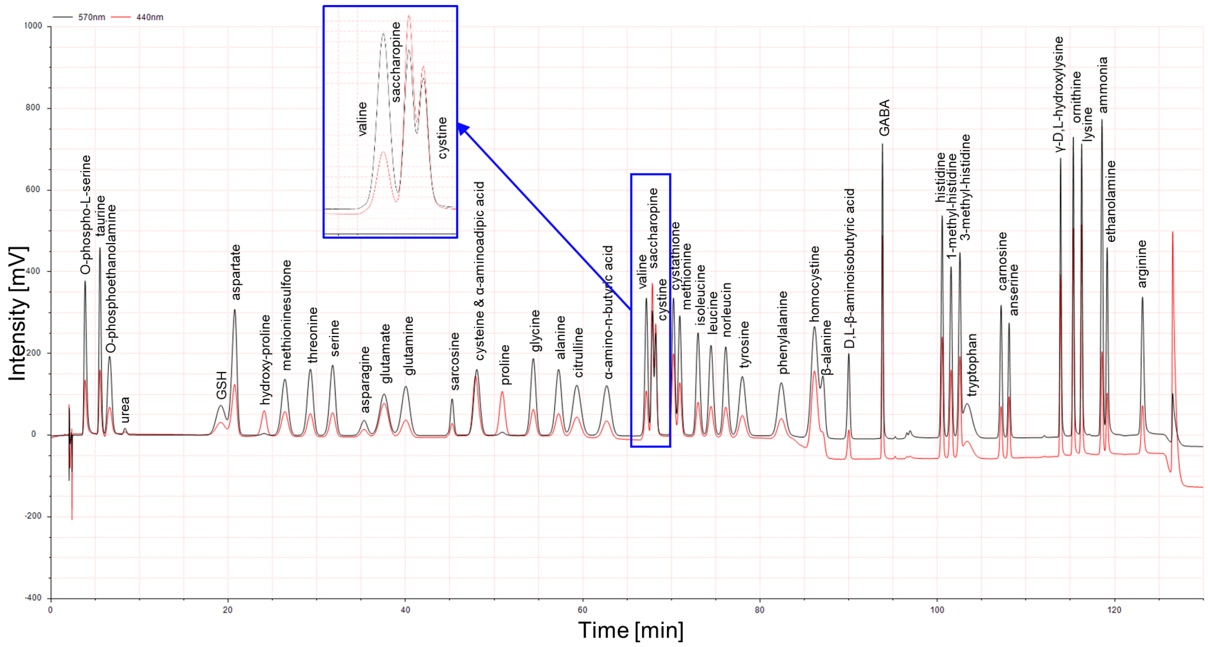

Supplementary Figure S4: Chromatogram of the amino acid standard including saccharopine (AAQ).

The chromatogram resulted from a calibration run with a mix of amino acid standards including 100 µM saccharopine. The standard analysis method was used here (not optimized for α-AAA) The blue boxes show a zoom-in on the area of interest. Each amino acid of the mixture had a concentration of 100 µM; except for cystine, which was 50 µM. Peaks are labeled with the respective amino acids. The chromatogram detected at an absorption of 570 nm is shown in black. The chromatogram recorded at 440 nm is shown in red. A volume of 100 µl was injected.

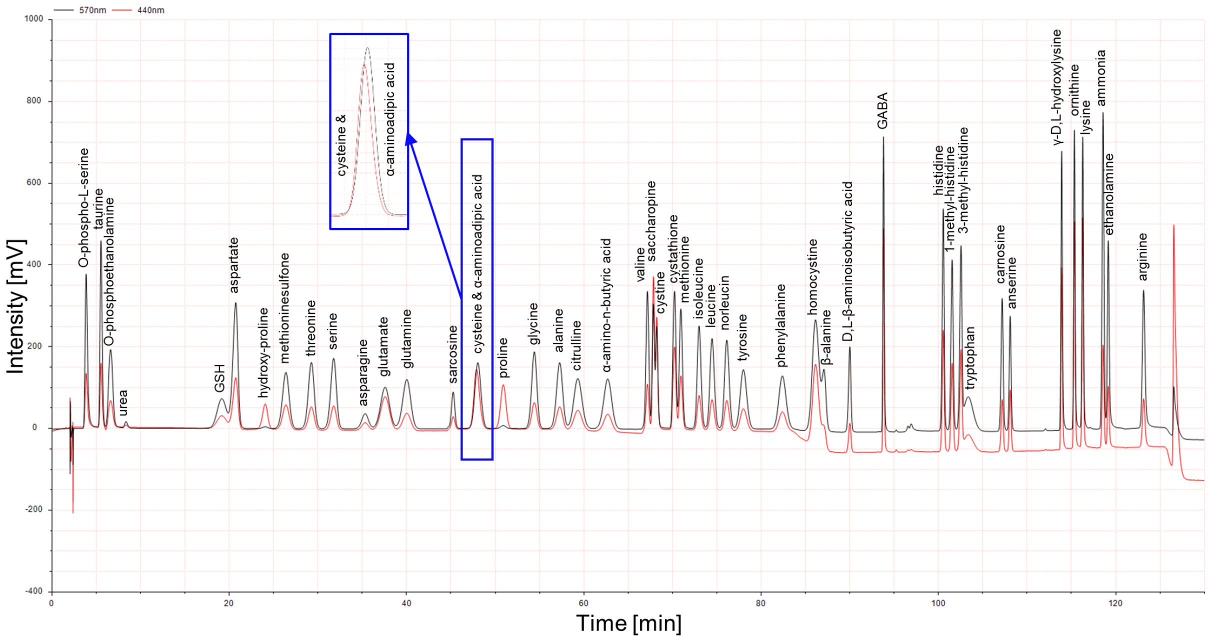

Supplementary Figure S5: Chromatogram of the amino acid standard highlighting the common peak of cysteine and α-aminoadipic acid with the standard method (AAQ).

The blue boxes show a zoom in on the common peak of cysteine and α-aminoadipic acid. Peaks are labeled with the respective amino acids.

Time [min]	Type of function	Parameter of function			
Initialisation	Composition of solvent	A: 75%	B: 25%	C: 0%	D: 0%
Initialisation	Flow rate	0.45 ml/min			
Initialisation	Upper limit of pressure	120 bar			
Initialisation	Amino flow rate	0.25 ml/min			
Initialisation	Amino reactor temperature	130 °C			
Initialisation	Amino column temperature	60 °C			
Initialisation	Amino reagent valve	Ninhydrin			
2	Amino auto zero				
10	Composition of solvent	A: 75%	B: 25%	C: 0%	D: 0%
20	Composition of solvent	A: 73%	B: 27%	C: 0%	D: 0%
22	Composition of solvent	A: 73%	B: 27%	C: 0%	D: 0%
22.1	Composition of solvent	A: 0%	B: 0%	C: 0%	D: 100%
22.1	Amino reagent valve	Wash (25% ethanol, 25% isopropanol)			
25	Composition of solvent	A: 0%	B: 0%	C: 0%	D: 100%
25	Amino column temperature	60 °C			
25.1	Composition of solvent	A: 75%	B: 25%	C: 0%	D: 0%
40	Composition of solvent	A: 75%	B: 25%	C: 0%	D: 0%
40	Amino column temperature	60 °C			

Supplementary Figure S6: Method used for the separation of cysteine and α-aminoadipic acid (AAQ2).

The time table indicates the changes in the buffer composition and the column temperature used for the sequential elution of cysteine and α-aminoadipic acid.

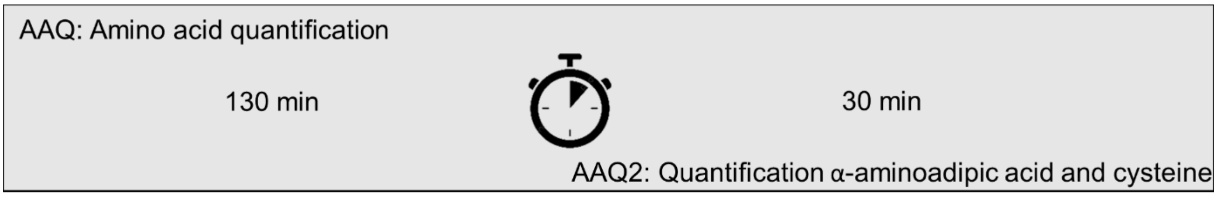

Metabolite	AAQ	AAQ2
O-phospho-L-serine	Detected	Not detected
Taurine	Detected	Not detected
O-phosphoethanolamine	Detected	Not detected
Urea	Detected	Not detected
GSH	Detected	Not detected
Aspartate	Detected	Not detected
Hydroxy-proline	Detected	Not detected
Methioninesulfone	Detected	Not detected
Threonine	Detected	Not detected
Serine	Detected	Not detected
Asparagine	Detected	Not detected
Glutamate	Detected	Not detected
Glutamine	Detected	Not detected
Sarcosine	Detected	Not detected
Cysteine	Combi peak	Detected
α-aminoadipic-acid		Detected
Proline	Detected	Not detected
Glycine	Detected	Not detected
Alanine	Detected	Not detected
Citrulline	Detected	Not detected
α-amino-n-butyric acid	Detected	Not detected
Valine	Detected	Not detected
Saccharopine	Detected	Not detected
Cystine	Detected	Not detected
Cystathione	Detected	Not detected
Methionine	Detected	Not detected
Isoleucine	Detected	Not detected
Leucine	Detected	Not detected
Norleucine	Detected	Not detected
Tyrosine	Detected	Not detected
Phenylalanine	Detected	Not detected
Homocystine	Detected	Not detected
β-alanine	Detected	Not detected
D,L,β-aminoisobutyric acid	Detected	Not detected
GABA	Detected	Not detected
Histidine	Detected	Not detected
1-methyl-histidine	Detected	Not detected
3-methyl-histidine	Detected	Not detected
Tryptophan	Detected	Not detected
Carnosine	Detected	Not detected
Anserine	Detected	Not detected
γ-D,L,-hydroxylysine	Detected	Not detected
Ornithine	Detected	Not detected
Lysine	Detected	Not detected
Ammonia	Detected	Not detected
Ethanolamine	Detected	Not detected
Arginine	Detected	Not detected

Supplementary Figure S7: Metabolites detected by AAQ and AAQ2

The AAQ method was setup to quantify 45 metabolites. One run took 130 min; cysteine and alpha-aminoadipic acid eluted as a combination peak, which did not allow quantification of its components. The AAQ2 method (runtime of 30 min) allowed a quantification of cysteine and alpha-aminoadipic acid.

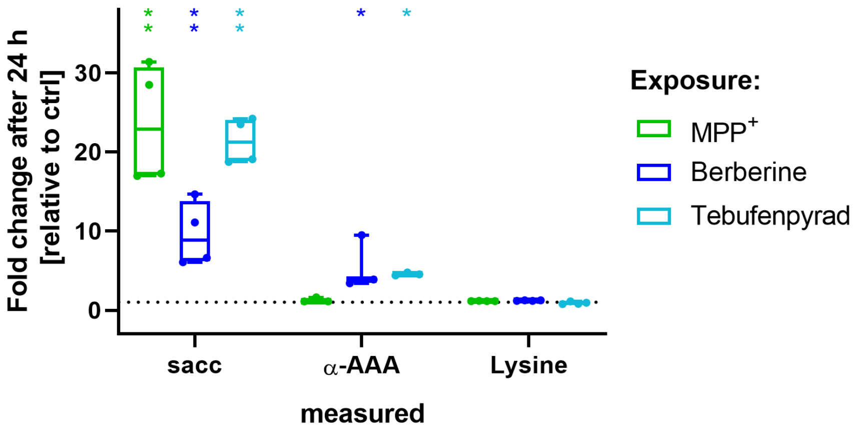

Supplementary Figure S8: Specific accumulation of the L-lysine degradation metabolites saccharopine and α-aminoadipic acid upon mitochondrial stress.

Pre-differentiated d2 LUHMES cells were seeded in differentiation medium and incubated at 37 °C, 5% CO_2 for 1 h to allow attachment before treatment with 10 µM berberine, 10 µM MPP^+ or 20 µM tebufenpyrad. After 24 h, cells were lysed and saccharopine (sacc), α-aminoadipic acid (α-AAA) and lysine were quantified. All data are normalized to the solvent controls and expressed as fold change.

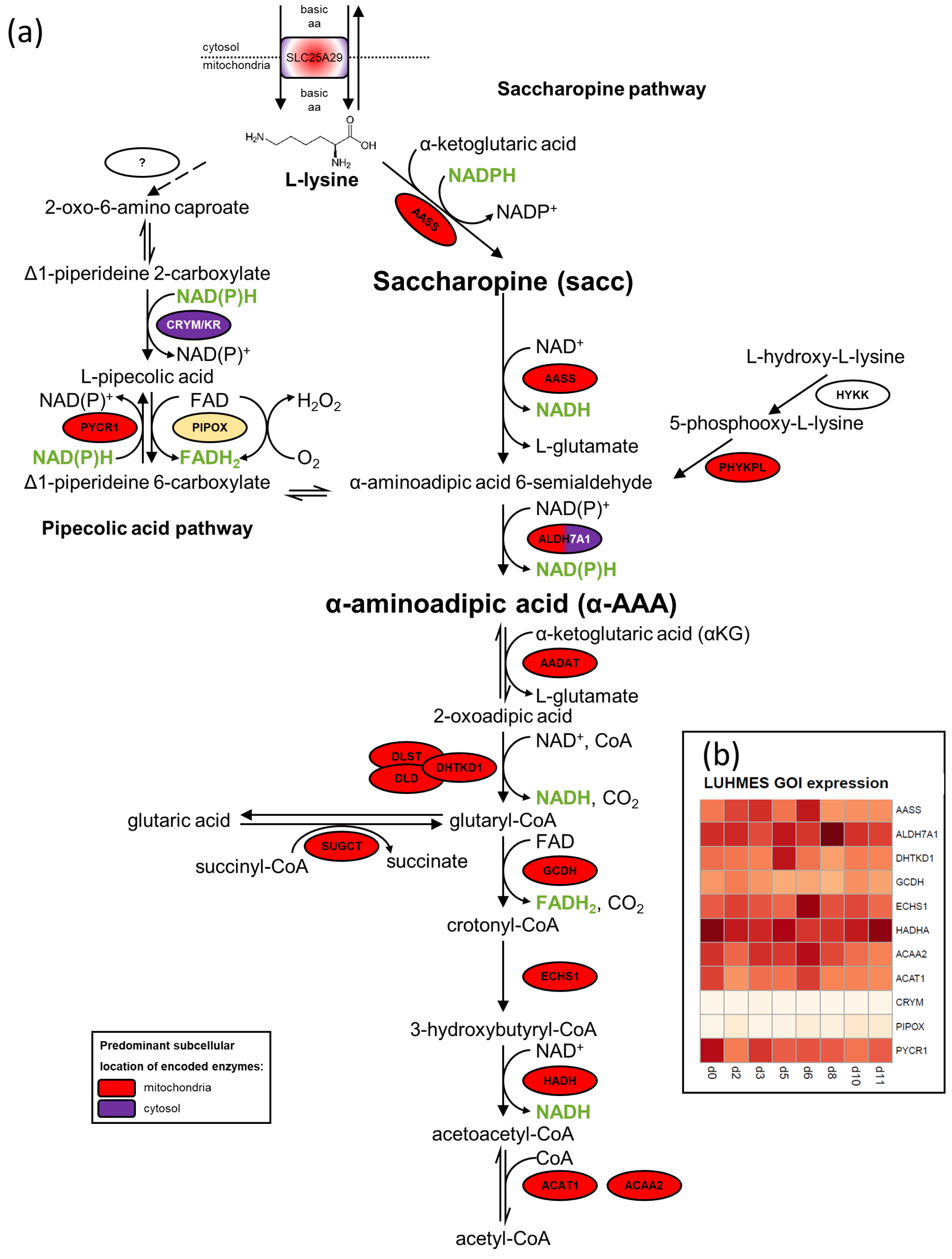

Supplementary Figure S9: Overview of the L-lysine degradation pathway and the expression of related genes enzymes in LUHMES cells.

(**a**) L-lysine may be degraded either via the mitochondrial saccharopine pathway or the pipecolic acid pathway. The two pathways converge at α-aminoadipic acid 6-semialdehyde, which can also be generated from L-hydroxy-L-lysine. The information was extracted from Leandro et. al [12]. (**b**) The gene expression profile of LUHMES cells was analyzed by TempOSeq sequencing (as described in Loser et al., 2021, reference 49). Samples were prepared from proliferating cells (d0) and at different days of differentiation (d2 - d11). Five biological replicates were analyzed per condition. Data was normalized to sequencing depth

and gene length before logarithmic transformation. The original set of data is provided in a supplementary raw data file (also available at Zenodo) [30]. The gene expression level is color-coded with white indicating no expression and full red signifying an expression level of ≥ 256 counts per million transcripts (cpm) [14]. **Abbreviations:** AADAT, kynurenine/-aminoadipic acid aminotransferase; AASS, 2-aminoadipic acid semiadehyde synthase; ACAA2, 3-ketoacyl-CoA thiolase; ACAT1, acetoacetyl-CoA thiolase; ALDH7A1, 2-aminoadipic acid semialdehyde dehydrogenase; CRYM/KR, -crystallin/ ketimine reductase; DHTKD1, dehydrogenase E1 and transketolase domain containing protein 1; DLD, dihydrolipoyl dehydrogenase; DLST, dihydrolipoyl succinyltransferase; ECHS1, enoyl-CoA hydratase; GCDH, glutaryl-CoA dehydrogenase; HADH, 3-hydroxyacyl-CoA dehydrogenase; HYKK, hydroxylysine kinase; PHYKPL, 5-phosphohydroxy-L-lysine phospho-lyase; PIPOX, peroxisomal sarcosine oxidase, also known as pipecolic acid oxidase; PYCR1, pyrroline-5-carboxylate reductase 1; SLC25A29, mitochondrial basic amino acid transporter; SUGCT, succinyl-CoA:glutarate-CoA transferase.

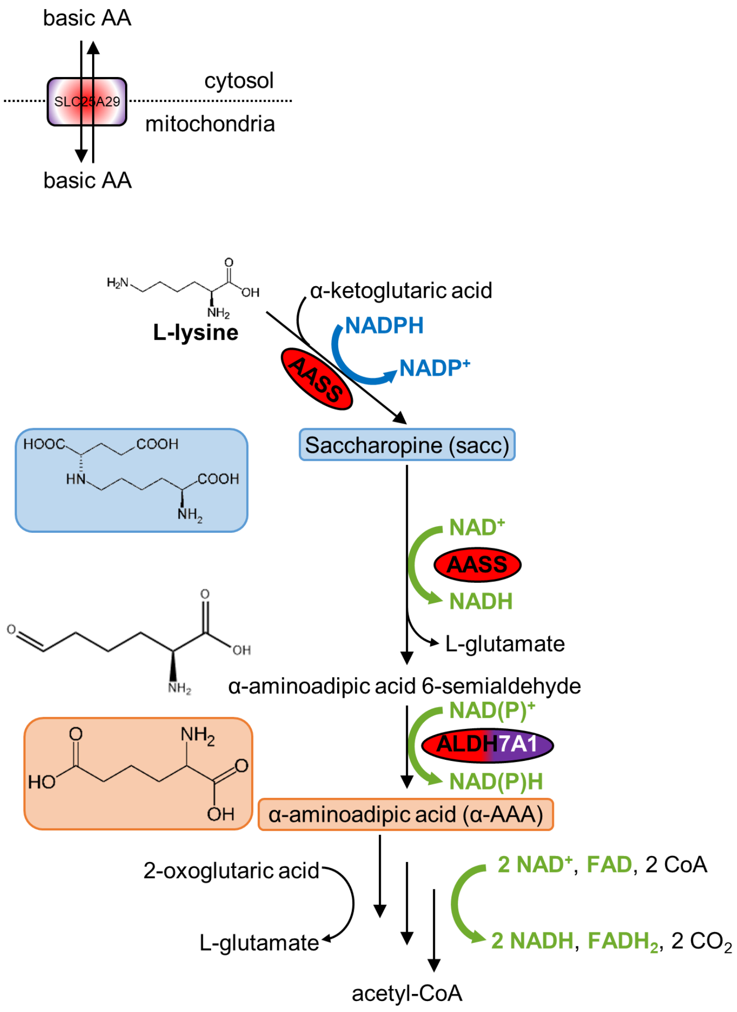

Supplementary Figure S10: The role of the cellular redox state in controlling the saccharopine pathway.

The initial part of the L-lysine degradation via the mitochondrial saccharopine pathway is shown. Amino acids are depicted with their structural formula in black. The metabolites of interest are highlighted in blue (saccharopine) and orange (α-aminoadipic acid). Mitochondrial enzymes are shown in red, cytosolic enzymes in purple. Reactions that further increase the cellular load with reduced nicotinamide nucleotides are indicated in green. Reactions that decrease the cellular load by oxidizing nicotinamide nucleotides are indicated in blue. **Abbreviations:** AASS, 2-aminoadipic acid semialdehyde synthase; ALDH7A1, 2-aminoadipic acid semialdehyde dehydrogenase.

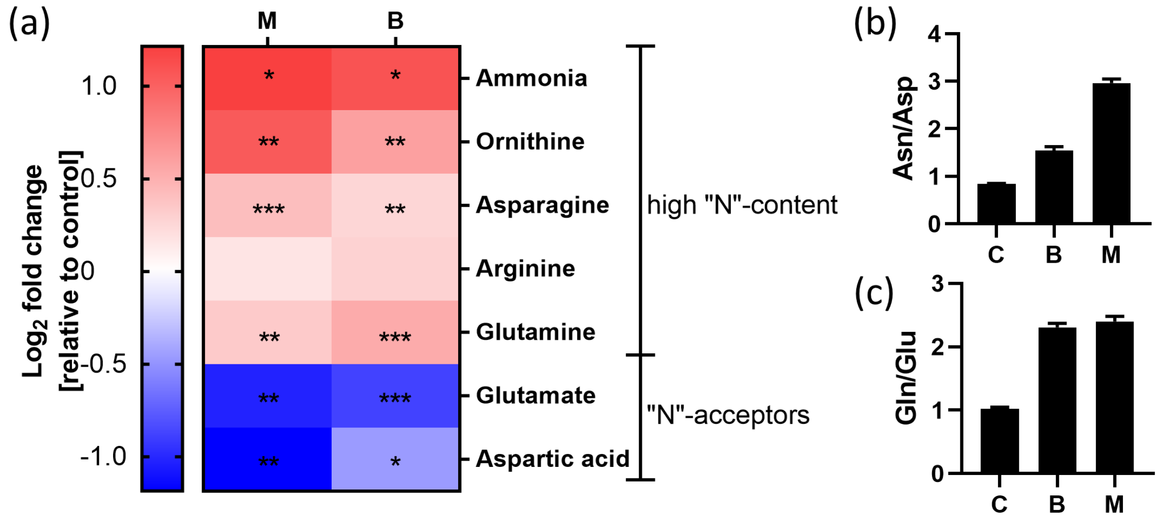

Supplementary Figure S11: cI inhibitors affect high "N"-content and "N"-acceptor amino acids

Pre-differentiated d2 LUHMES cells were seeded in differentiation medium and incubated at 37 °C, 5% CO_2 for 1 h. They were treated with solvent (C), 10 µM berberine (B) or 10 µM MPP^+ (M) for 24 h, before cells were lysed and their amino acid composition was analyzed. AAQ and AAQ2 were used to quantify metabolites in cell lysates. (**a**) All data are normalized to the solvent controls (C) and expressed as log2 fold change in a heat map. Negative values (blue) show a decrease of the free amino acid concentration compared to the solvent control; positive values (red) indicate an elevation of the amino acid concentration. All data are compiled in the supplementary excel file. For statistical analysis, treated samples were compared to the respective control using a mixed model ANOVA (amino acid x treatment) followed by Dunnett's multiple comparisons post hoc test. Significance levels are indicated by asterisks (*: adjusted $p \leq 0.05$; **: adjusted $p \leq 0.01$; ***: adjusted $p \leq 0.001$). (**b,c**) ratios have been calculated from the averaged absolute levels of aspartate (Asp) and asparagine (Asn) or glutamate (Glu) and glutamine (Gln) for better visualization of shifted relative amounts of the nitrogen donor/acceptor pairs. Data are displayed as means ± SD. Accordingly (**a**) visualizes how amino acids with high nitrogen (N) content increased, while typical N-acceptors decreased.

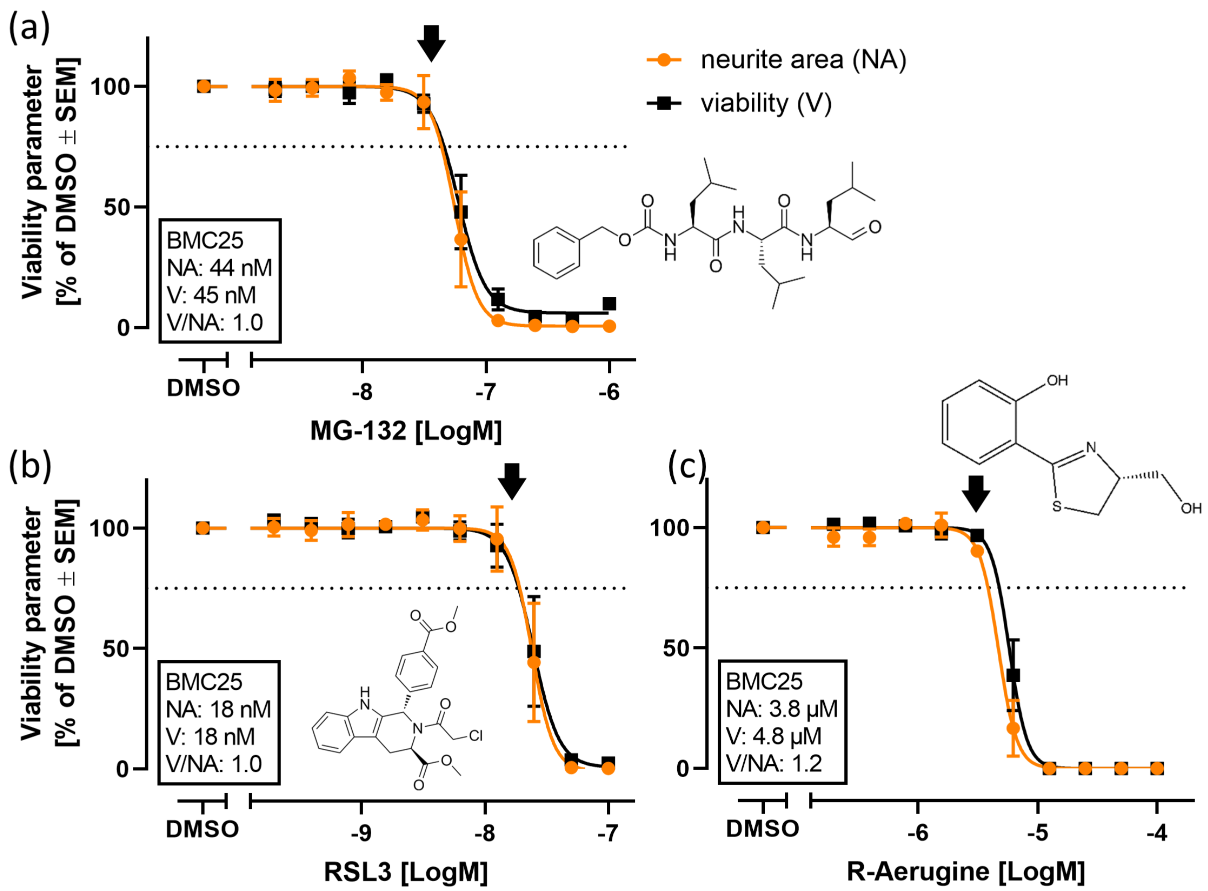

Supplementary Figure S12: Assessment of viability and neurite outgrowth in the presence of various toxicants.

Pre-differentiated d2 LUHMES cells were seeded in differentiation medium and incubated at 37 °C, 5% CO_2 for 1 h to ensure attachment prior to treatment. Cells were exposed to serial dilutions of (**a**) MG-132, (**b**) RSL3 or (**c**) R-aerugine (structural formulas are given). The cell viability (V; black) and neurite area (NA; orange) were assessed 24 h later by calcein-AM and H-33342 staining and high content imaging. Data are presented as means ± SEM (3-5 biological with three technical replicates). The benchmark concentrations at which the parameters are decreased by 25% (BMC25; dotted line) are given in black boxes.

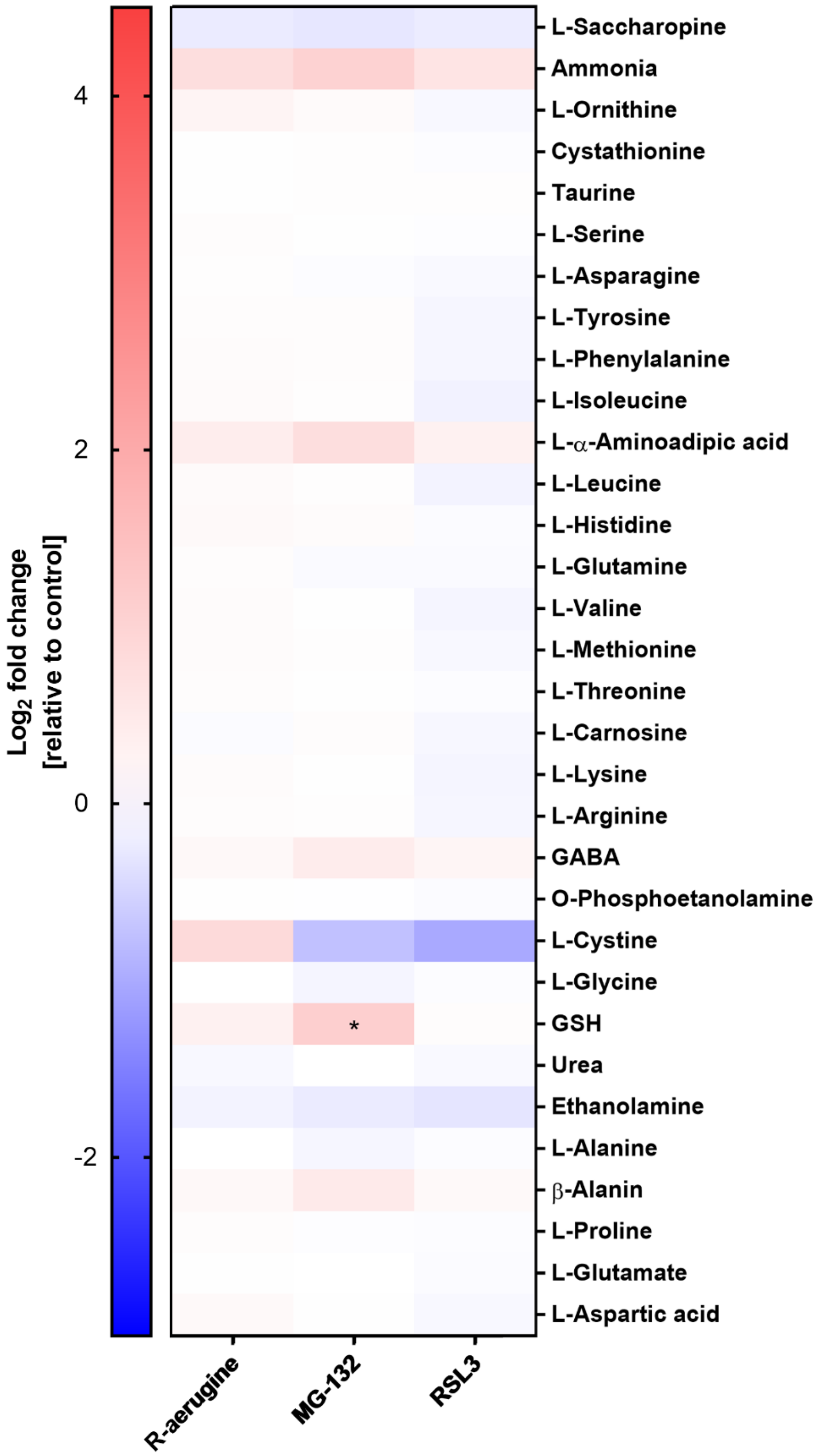

Supplementary Figure S13: Toxicant-induced changes of intracellular amino acids and related compounds by aerugine, MG-132 and RSL3

Pre-differentiated d2 LUHMES cells were seeded in differentiation medium and incubated at 37°C, 5% CO_2 for 1 h. They were treated with 3 µM R-aerugine, 30 nM MG-132 or 30 nM RSL3 for 24 h, before cells were lysed and their amino acid composition was analyzed. AAQ and AAQ2 were used to quantify metabolites in cell lysates. Based on the raw data, the log2-fold changes relative to the untreated control were calculated. All data are normalized to the solvent controls and expressed as log2 fold change in a heat map. Negative values (blue) show

a decrease of the free amino acid concentration compared to the control, positive values (red) indicate an elevation of the amino acid concentration. All data are compiled in the supplementary excel file. For statistical analysis, treated samples were compared to the respective control using a mixed model ANOVA (amino acid x treatment) followed by Dunnett's multiple comparisons post hoc test. Significance levels are indicated by asterisks (*: adjusted $p \leq 0.05$). Note that this data set is comparable (same technical/experimental approach, different compounds) to the data for cI inhibitors in Figure 6. Note also that the data displayed here refer to intracellular amino acids, while the data displayed in Figure 7 refer to changes of amino acid levels in the cell culture medium. For comparisons of MG-132 data to previous studies (describing several amino acid changes in LUHMES cells) it is important that here a low concentration of MG-132 was used that did not kill cells (highest non-cytotoxic concentration), while earlier studies used a concentration that was lethal.

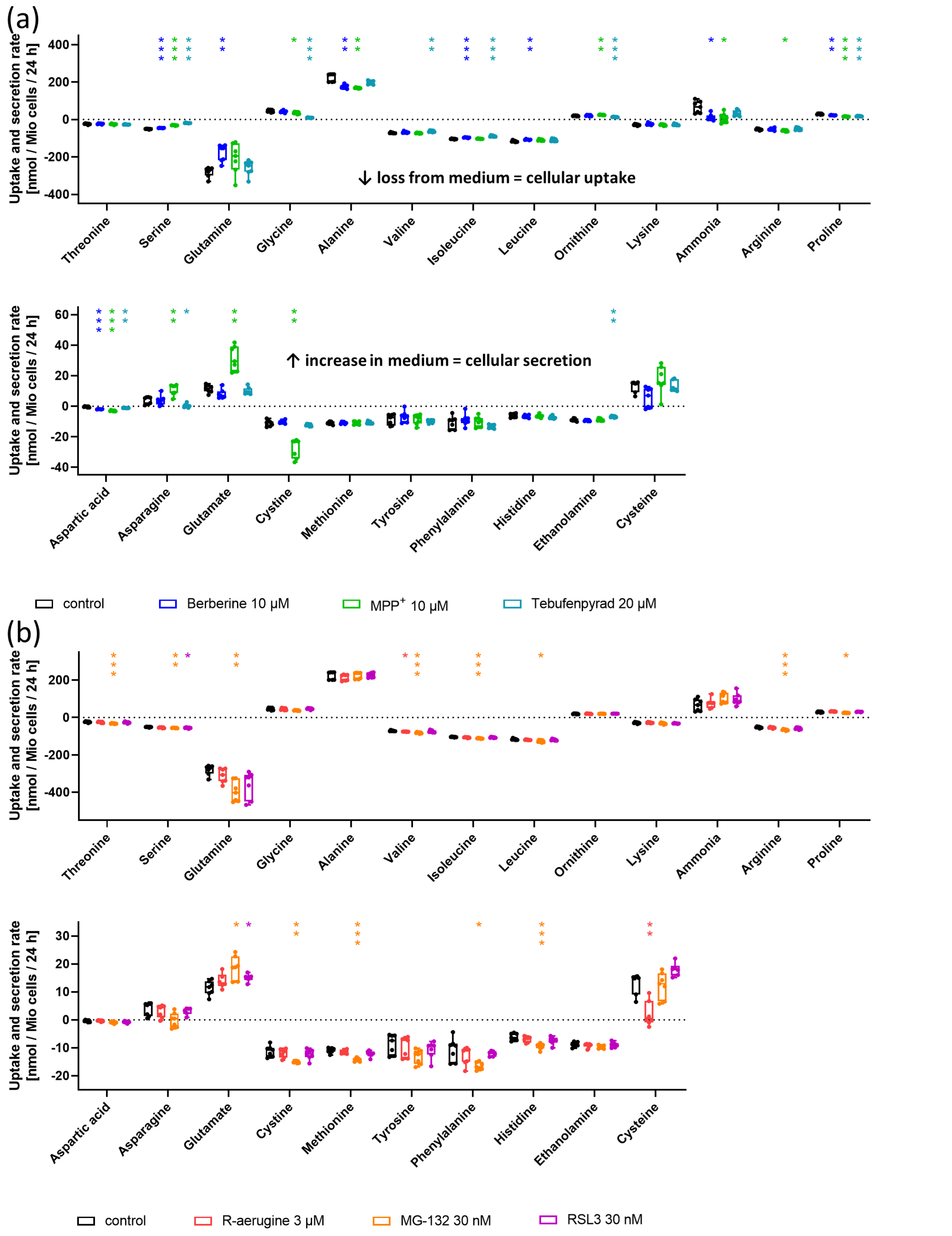

Supplementary Figure S14: Amino acid uptake and secretion rates of LUHMES cells after treatment with various toxicants.

Predifferentiated d2 LUHMES cells were seeded in differentiation medium and incubated at 37 °C, 5% CO_2 for 1 h, to ensure attachement of the cells prior to treatment. (**a**) Cells were treated with 10 µM berberine (blue), 10 µM MPP^+ (green) or 20 µM tebufenpyrad (turquoise) for 24 h before the amino acid composition of the medium was analyzed. (**b**) Cells were treated with 3

µM R-aerugine (red), 30 nM MG-132 (orange) or 30 nM RSL3 (purple) for 24 h before analyzing the amino acid composition of the medium.

Results of untreated control cells are shown in black. Uptake and secretion rates were calculated from the difference of the measured amino acid concentrations in medium with and without cells. Negative values represent cellular uptake of amino acids (= net loss from medium), positive values indicate a net secretion. Data are presented as box-whisker plots with a total of seven measured replicates obtained from two separate experiments. For statistical analysis, treated samples were compared to the respective control using a mixed model ANOVA (amino acid x sugar) followed by Dunnett's multiple comparisons post hoc test. Significance levels are indicated by asterisks (*: adjusted $p \leq 0.05$; **: adjusted; $p \leq 0.01$; ***: adjusted $p \leq 0.001$).

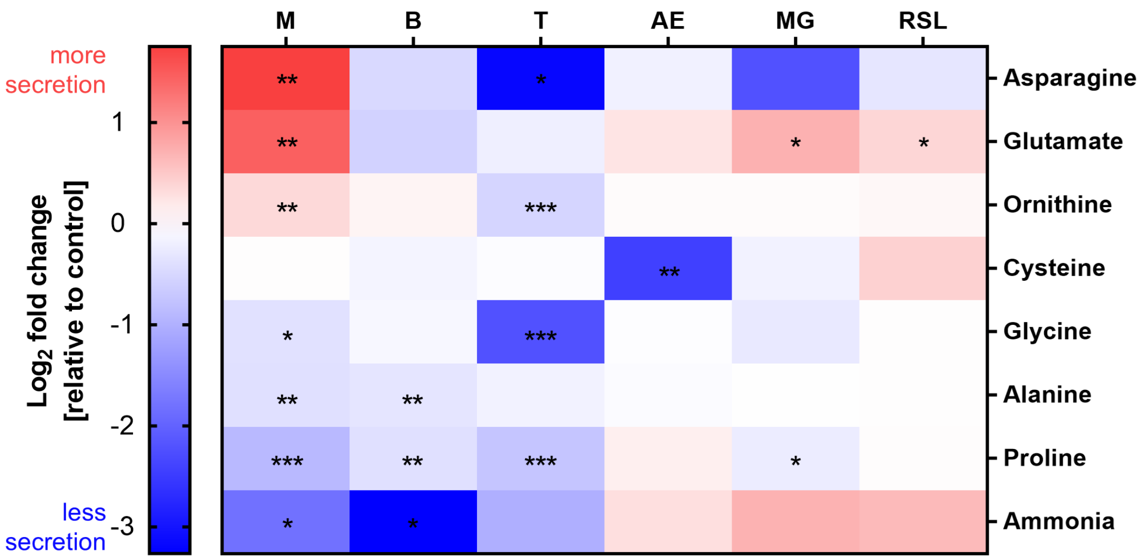

Supplementary Figure S15: Changes in amino acid secretion rates of LUHMES cells after exposure to various toxicants.

Pre-differentiated d2 LUHMES cells were seeded as in figure 6 and treated with 10 µM MPP$^+$ (M), 10 µM berberine (B), 20 µM tebufenpyrad (T), 3 µM R-aerugine (AE), 30 nM MG-132 (MG) or 30 nM RSL3 (RSL) for 24 h, before the amino acid composition of the medium was analyzed. All data are normalized to the secretion rate of solvent-treated cells and expressed as log2 fold change. The heat map displays all amino acid secretion rates changed significantly by at least one of the toxicants. Negative values (blue) show a decrease of secretion rate, positive values (red) indicate an increased secretion. For statistical analysis, treated samples were compared to the respective control using a mixed model ANOVA (amino acid x treatment) followed by Dunnett's multiple comparisons post hoc test. Significance levels are indicated by asterisks (*: adjusted $p \leq 0.05$; **: adjusted $p \leq 0.01$; ***: adjusted $p \leq 0.001$). Note that (e.g. for MPP$^+$) asparagine is both increased in cells (Figure 6) and in the medium (increased secretion), which is a clear indication of the excess of this amino acid. In the case of ammonia, there is an increase in cells (Figure 6), but a reduced secretion. This situation is more complex and possibly no steady state has been reached.

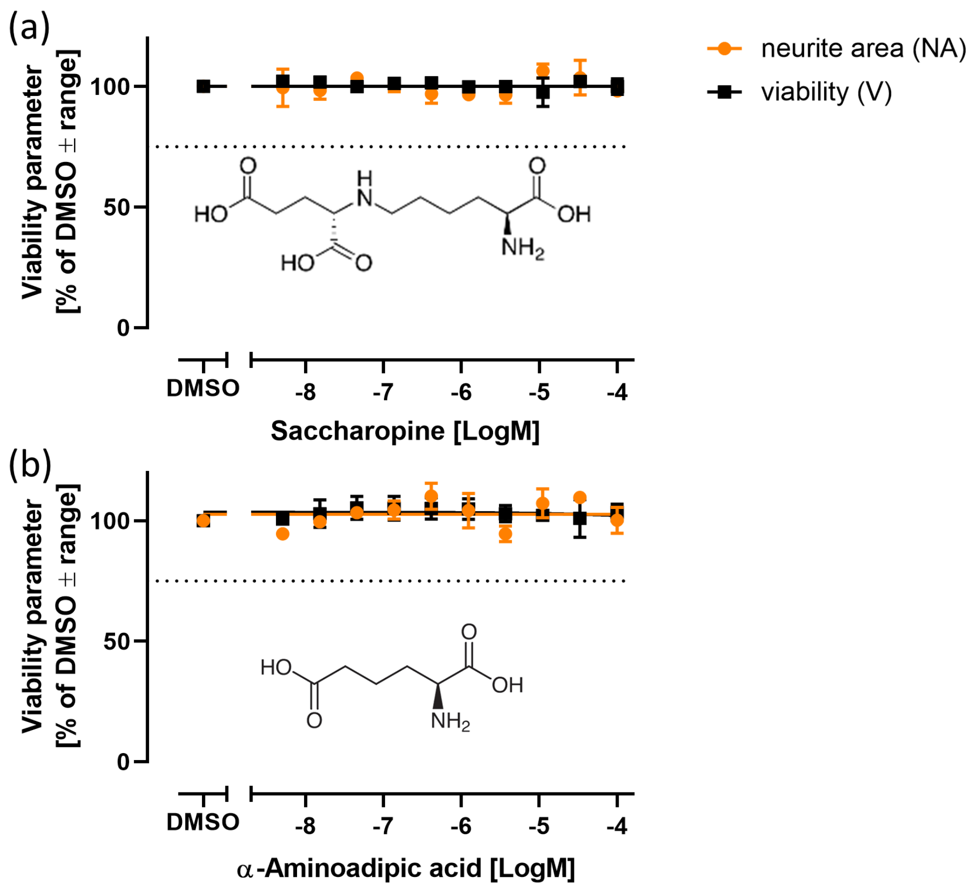

Supplementary Figure S16: Assessment of viability and neurite outgrowth in the presence of L-lysine degradation products.

Pre-differentiated d2 LUHMES cells were seeded in differentiation medium and incubated at 37 °C, 5% CO_2 for 1 h to ensure attachment prior to treatment. Cells were exposed to serial dilutions of (**a**) saccharopine or (**b**) α-aminoadipic acid (chemical formulas are given). The cell viability (V; black) and neurite area (NA; orange) were assessed 24 h later by calcein-AM and H-33342 staining and high content imaging. Data are presented as means ± range of two biological replicates (each based on three technical replicates).

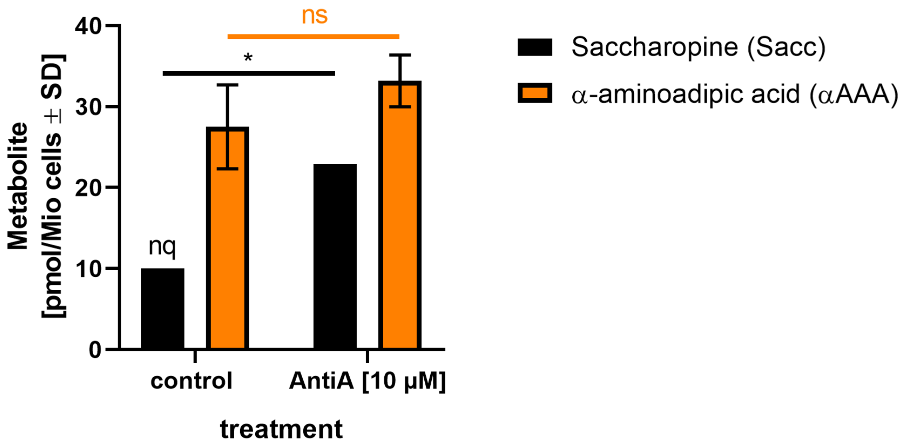

Supplementary Figure S17: cIII-inhibition by Antimycin A increases the intracellular saccharopine content

Pre-differentiated d2 LUHMES cells were seeded as in Figure 6. Cells were treated with 10 µM antimycin A (AntiA). Saccharopine (Sacc) and α-aminoadipic acid (α-AAA) were quantified. Data are expressed as average ± SD of 2 independently-prepared samples. Low levels of saccharopine were detectable under the control conditions, but the level was too low to quantify (nq: not quantifiable). The peak upper limit shows the quantification limit. For statistical analysis, treated samples were compared to the respective control using a 2-way ANOVA followed by Dunnett's multiple comparisons post hoc test. Significance levels are indicated by asterisks (*:p $\leq$ 0.05; ns: not significant).

Supplementary references

All references are also in the main manuscript reference list. Note that they have differernt numbers in that list, according to the position where they are cited in the main text. The numbering here referes to citatrioins within the supplementary material.

1. Mainka, T.; Fischer, J.F.; Huebl, J.; Jung, A.; Lier, D.; Mosejova, A.; Skorvanek, M.; de Koning, T.J.; Kuhn, A.A.; Freisinger, P.; et al. The neurological and neuropsychiatric spectrum of adults with late-treated phenylketonuria. *Parkinsonism Relat Disord* **2021**, *89*, 167-175, doi:10.1016/j.parkreldis.2021.06.011.
2. van Wegberg, A.M.J.; MacDonald, A.; Ahring, K.; Belanger-Quintana, A.; Blau, N.; Bosch, A.M.; Burlina, A.; Campistol, J.; Feillet, F.; Gizewska, M.; et al. The complete European guidelines on phenylketonuria: diagnosis and treatment. *Orphanet J Rare Dis* **2017**, *12*, 162, doi:10.1186/s13023-017-0685-2.
3. de Groot, M.J.; Hoeksma, M.; Blau, N.; Reijngoud, D.J.; van Spronsen, F.J. Pathogenesis of cognitive dysfunction in phenylketonuria: review of hypotheses. *Mol Genet Metab* **2010**, *99 Suppl 1*, S86-89, doi:10.1016/j.ymgme.2009.10.016.
4. Van Hove, J.L.K.; Coughlin, C., II; Swanson, M.; Hennermann, J.B. Nonketotic Hyperglycinemia. In *GeneReviews(®)*, Adam, M.P., Bick, S., Mirzaa, G.M., Pagon, R.A., Wallace, S.E., Amemiya, A., Eds.; University of Washington, Seattle: Seattle (WA), 1993.
5. Escande-Beillard, N.; Loh, A.; Saleem, S.N.; Kanata, K.; Hashimoto, Y.; Altunoglu, U.; Metoska, A.; Grandjean, J.; Ng, F.M.; Pomp, O.; et al. Loss of PYCR2 Causes Neurodegeneration by Increasing Cerebral Glycine Levels via SHMT2. *Neuron* **2020**, *107*, 82-94.e86, doi:10.1016/j.neuron.2020.03.028.
6. Zhang, S.; Zeng, X.; Ren, M.; Mao, X.; Qiao, S. Novel metabolic and physiological functions of branched chain amino acids: a review. *J Anim Sci Biotechnol* **2017**, *8*, 10, doi:10.1186/s40104-016-0139-z.
7. Strauss, K.A.; Morton, D.H. Branched-chain Ketoacyl Dehydrogenase Deficiency: Maple Syrup Disease. *Curr Treat Options Neurol* **2003**, *5*, 329-341, doi:10.1007/s11940-003-0039-3.
8. Farias, H.R.; Gabriel, J.R.; Cecconi, M.L.; Lemos, I.S.; de Rezende, V.L.; Wessler, L.B.; Duarte, M.B.; Scaini, G.; de Oliveira, J.; Streck, E.L. The metabolic effect of α-ketoisocaproic acid: in vivo and in vitro studies. *Metab Brain Dis* **2021**, *36*, 185-192, doi:10.1007/s11011-020-00626-y.
9. Paul, B.D.; Sbodio, J.I.; Xu, R.; Vandiver, M.S.; Cha, J.Y.; Snowman, A.M.; Snyder, S.H. Cystathionine γ-lyase deficiency mediates neurodegeneration in Huntington's disease. *Nature* **2014**, *509*, 96-100, doi:10.1038/nature13136.
10. Bonetti, F.; Brombo, G.; Zuliani, G. The relationship between hyperhomocysteinemia and neurodegeneration. *Neurodegener Dis Manag* **2016**, *6*, 133-145, doi:10.2217/nmt-2015-0008.
11. Lei, C.; Lv, Z.; Ran, Q.; Jiang, F.; Zhang, M. Homocysteine and diabetic retinopathy. *Exp Eye Res* **2026**, *262*, 110729, doi:10.1016/j.exer.2025.110729.
12. Leandro, J.; Houten, S.M. The lysine degradation pathway: Subcellular compartmentalization and enzyme deficiencies. *Molecular Genetics and Metabolism* **2020**, *131*, 14-22, doi:https://doi.org/10.1016/j.ymgme.2020.07.010.
13. Crowther, L.M.; Mathis, D.; Poms, M.; Plecko, B. New insights into human lysine degradation pathways with relevance to pyridoxine-dependent epilepsy due to antiquitin deficiency. *J Inherit Metab Dis* **2019**, *42*, 620-628, doi:10.1002/jimd.12076.
14. Loser, D.; Schaefer, J.; Danker, T.; Moller, C.; Brull, M.; Suciu, I.; Uckert, A.K.; Klima, S.; Leist, M.; Kraushaar, U. Human neuronal signaling and communication assays to assess functional neurotoxicity. *Arch Toxicol* **2021**, *95*, 229-252, doi:10.1007/s00204-020-02956-3.
15. Suciu, I.; Delp, J.; Gutbier, S.; Suess, J.; Henschke, L.; Celardo, I.; Mayer, T.U.; Amelio, I.; Leist, M. Definition of the Neurotoxicity-Associated Metabolic Signature Triggered by Berberine

and Other Respiratory Chain Inhibitors. *Antioxidants (Basel)* **2023**, *13*, doi:10.3390/antiox13010049.
16. Ye, L.; Park, J.J.; Peng, L.; Yang, Q.; Chow, R.D.; Dong, M.B.; Lam, S.Z.; Guo, J.; Tang, E.; Zhang, Y.; et al. A genome-scale gain-of-function CRISPR screen in CD8 T cells identifies proline metabolism as a means to enhance CAR-T therapy. *Cell Metab* **2022**, *34*, 595-614.e514, doi:10.1016/j.cmet.2022.02.009.
17. Moreau, R.; Clària, J.; Aguilar, F.; Fenaille, F.; Lozano, J.J.; Junot, C.; Colsch, B.; Caraceni, P.; Trebicka, J.; Pavesi, M.; et al. Blood metabolomics uncovers inflammation-associated mitochondrial dysfunction as a potential mechanism underlying ACLF. *J Hepatol* **2020**, *72*, 688-701, doi:10.1016/j.jhep.2019.11.009.
18. McPhail, M.J.W.; Coen, M. Metabolomics to discriminate between acute decompensation and acute-on-chronic liver failure in cirrhosis. *J Hepatol* **2020**, *73*, 730-732, doi:10.1016/j.jhep.2020.03.021.
19. Zhou, J.; Wang, X.; Wang, M.; Chang, Y.; Zhang, F.; Ban, Z.; Tang, R.; Gan, Q.; Wu, S.; Guo, Y.; et al. The lysine catabolite saccharopine impairs development by disrupting mitochondrial homeostasis. *J Cell Biol* **2019**, *218*, 580-597, doi:10.1083/jcb.201807204.
20. Leandro, J.; Houten, S.M. Saccharopine, a lysine degradation intermediate, is a mitochondrial toxin. *J Cell Biol* **2019**, *218*, 391-392, doi:10.1083/jcb.201901033.
21. Beier, U.H.; Hartung, E.A.; Concors, S.; Hernandez, P.T.; Wang, Z.; Perry, C.; Baur, J.A.; Denburg, M.R.; Hancock, W.W.; Gade, T.P.; et al. Tissue metabolic profiling shows that saccharopine accumulates during renal ischemic-reperfusion injury, while kynurenine and itaconate accumulate in renal allograft rejection. *Metabolomics* **2020**, *16*, 65, doi:10.1007/s11306-020-01682-2.
22. Carson, N.A.; Scally, B.G.; Neill, D.W.; Carre, L.J. Saccharopinuria: a new inborn error of lysine metabolism. *Nature* **1968**, *218*, 679, doi:10.1038/218679a0.
23. Garcia-Bermudez, J.; Baudrier, L.; La, K.; Zhu, X.G.; Fidelin, J.; Sviderskiy, V.O.; Papagiannakopoulos, T.; Molina, H.; Snuderl, M.; Lewis, C.A.; et al. Aspartate is a limiting metabolite for cancer cell proliferation under hypoxia and in tumours. *Nat Cell Biol* **2018**, *20*, 775-781, doi:10.1038/s41556-018-0118-z.
24. Fu, A.; Danial, N.N. Grasping for aspartate in tumour metabolism. *Nat Cell Biol* **2018**, *20*, 738-739, doi:10.1038/s41556-018-0137-9.
25. Sullivan, L.B.; Luengo, A.; Danai, L.V.; Bush, L.N.; Diehl, F.F.; Hosios, A.M.; Lau, A.N.; Elmiligy, S.; Malstrom, S.; Lewis, C.A.; et al. Aspartate is an endogenous metabolic limitation for tumour growth. *Nat Cell Biol* **2018**, *20*, 782-788, doi:10.1038/s41556-018-0125-0.
26. Sullivan, L.B.; Gui, D.Y.; Hosios, A.M.; Bush, L.N.; Freinkman, E.; Vander Heiden, M.G. Supporting Aspartate Biosynthesis Is an Essential Function of Respiration in Proliferating Cells. *Cell* **2015**, *162*, 552-563, doi:10.1016/j.cell.2015.07.017.
27. Birsoy, K.; Wang, T.; Chen, W.W.; Freinkman, E.; Abu-Remaileh, M.; Sabatini, D.M. An Essential Role of the Mitochondrial Electron Transport Chain in Cell Proliferation Is to Enable Aspartate Synthesis. *Cell* **2015**, *162*, 540-551, doi:10.1016/j.cell.2015.07.016.
28. Wahl, D.R.; Byersdorfer, C.A.; Ferrara, J.L.; Opipari, A.W., Jr.; Glick, G.D. Distinct metabolic programs in activated T cells: opportunities for selective immunomodulation. *Immunol Rev* **2012**, *249*, 104-115, doi:10.1111/j.1600-065X.2012.01148.x.
29. Geiger, R.; Rieckmann, J.C.; Wolf, T.; Basso, C.; Feng, Y.; Fuhrer, T.; Kogadeeva, M.; Picotti, P.; Meissner, F.; Mann, M.; et al. L-Arginine Modulates T Cell Metabolism and Enhances Survival and Anti-tumor Activity. *Cell* **2016**, *167*, 829-842.e813, doi:10.1016/j.cell.2016.09.031.
30. Anna-Katharina Ückert, A.L., Regina Pape, Mahshid Alimohammadi, Ivano Amelio, Marcel Leist. Raw data for the study: Saccharopine and α-aminoadipic acid as biomarkers for mitochondrial stress in neurons. **2026**, doi:https://doi.org/10.5281/zenodo.21824967.
